# miR‐140‐5p Overexpression Contributes to Oxidative Stress and Mitochondrial Dysfunction in Hutchinson‐Gilford Progeria Syndrome Fibroblasts Through NRF2 Pathway

**DOI:** 10.1111/acel.70276

**Published:** 2025-10-31

**Authors:** Léa Toury, Diane Frankel, Sara Nael, Mario Abaji, Léa Le Goff, Marie Basset, Coraline Airault, Bertrand Vernay, Elva María Novoa‐del‐Toro, Catherine Bartoli, Anaïs Baudot, Frederique Magdinier, Elise Kaspi, Patrice Roll

**Affiliations:** ^1^ Aix Marseille Université, INSERM, MMG Marseille France; ^2^ Aix Marseille Université, APHM, INSERM MMG, Service de Biologie Cellulaire, Hôpital la Timone Marseille France; ^3^ Aix Marseille Université, APHM, INSERM MMG, M2GM, Hôpital la Timone Marseille France; ^4^ Aix Marseille Université, CNRS INP UMR7051, Neuro‐Cellular Imaging Service and Nikon Center for Neuro‐NanoImaging Marseille France

**Keywords:** Hutchinson‐Gilford progeria syndrome, lamins, microRNA, miR‐140, mitochondria, NRF2, oxidative stress

## Abstract

Hutchinson‐Gilford Progeria Syndrome (HGPS) is a rare, fatal genetic disorder characterized by accelerated aging. The accumulation of an abnormal and toxic protein called progerin within HGPS nuclei disrupts cellular processes, including gene expression and DNA repair. Oxidative stress, resulting from an imbalance between reactive oxygen species (ROS) production and antioxidant defense, is one of the hallmarks of HGPS. To identify novel molecular mechanisms underlying HGPS pathogenesis, we performed miRNA expression profiling in HGPS compared to healthy control fibroblasts. We identified 10 differentially expressed (DE) miRNAs between HGPS and control cells. We focused on miR‐140‐5p and miR‐140‐3p, 2 miRNAs upregulated in HGPS fibroblasts. miR‐140‐5p is known to directly target the transcript of *NRF2*, a master regulator of the antioxidant response. Using in vitro mimic and antimiR transfections, we demonstrated that miR‐140‐5p overexpression in HGPS fibroblasts results in the downregulation of the NRF2/KEAP1/HO‐1 antioxidant pathway, leading to increased oxidative stress. Furthermore, our results indicate that miR‐140‐5p overexpression induces mitochondrial dysfunction, characterized by a reduced oxidative phosphorylation capacity and affects other hallmarks of aging. By targeting regulation of oxidative stress and mitochondrial function through NRF2, miR‐140‐5p may play a pivotal role in the pathophysiology of HGPS and open new therapeutic avenues.

## Graphical abstract

This study identifies a novel molecular mechanism involving miR‐140‐5p that contributes to the pathogenesis of HGPS. By decreasing *NRF2* expression, miR‐140‐5p overexpression results in downregulation of the NRF2/KEAP1/HO‐1 antioxidant pathway in HGPS fibroblasts, leading to increased oxidative stress and mitochondrial dysfunction, two hallmarks of aging. (Figure created in BioRender. Magdinier, F. (2025) https://BioRender.com/zixfr1g).

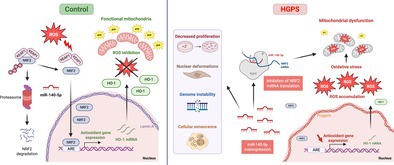

## 1 Introduction

Hutchinson‐Gilford Progeria Syndrome (HGPS; OMIM #176670) is a devastating rare genetic disorder characterized by accelerated aging beginning in childhood. Children with HGPS exhibit a multisystemic disease, presenting with a wide range of premature aging phenotypes, including growth retardation, hair loss, lipoatrophy, amyotrophy, skeletal abnormalities, and cardiovascular disease. Tragically, they succumb to these complications around 14 years old, usually due to myocardial infarction or stroke as a result of early atherosclerosis (Gordon et al. 1993).

The underlying cause of HGPS lies in a mutation within the *LMNA* gene, which encodes Lamin A and Lamin C, crucial proteins that, along with Lamins B, constitute the nuclear lamina, involved in nuclear integrity and function. The most common form of HGPS arises from a de novo heterozygous mutation (c.1824C>T, p.G608G) in exon 11 of the *LMNA* gene. This mutation leads to aberrant splicing that results in the production of a truncated and toxic nuclear lamin A precursor (prelamin AΔ50), called progerin (De Sandre‐Giovannoli et al. 2003; Eriksson et al. 2003).

HGPS belongs to a broader category of diseases called laminopathies, all of which involve defects in lamins or their interacting proteins. Other, rarer forms of premature aging syndromes, collectively termed HGPS‐like, share many clinical features with HGPS. These conditions arise from diverse de novo exonic or intronic mutations that also result in the intracellular accumulation of toxic prelamin A isoforms. The severity of the HGPS‐like phenotype can vary depending on the specific mutation, ranging from a mild adult form to a severe neonatal form of progeria (Barthélémy et al. 2015). The accumulation of progerin or other toxic prelamin A isoforms within the nucleus disrupts the nuclear lamina, triggering a cascade of cellular defects, including nuclear envelope abnormalities, loss of heterochromatin, DNA damage, and oxidative stress (Cau et al. 2014; Gonzalo et al. 2017). All these defects lead to premature cell senescence that contributes to accelerated aging.

Oxidative stress, resulting from an imbalance between reactive oxygen species (ROS) production and antioxidant defense mechanisms, is a hallmark of HGPS and a major contributor to its devastating effects (Cau et al. 2014). Chronic exposure to excessive ROS can lead to irreversible cellular damage through the oxidation of proteins, lipids, and DNA. In healthy cells, a finely tuned balance exists between ROS generation and removal by antioxidant mechanisms. However, in HGPS cells, this balance is disrupted. Increased ROS production coupled with insufficient detoxification by the antioxidant defense system leads to a state of chronic oxidative stress (Kristiani and Kim 2023). Consequently, fibroblasts of young HGPS patients exhibit significantly higher levels of oxidized proteins compared to age‐matched controls, resembling those found in 80‐year‐old individuals (Trigueros‐Motos et al. 2011; Viteri et al. 2010).

The nuclear factor erythroid 2‐related factor 2 (NRF2) pathway plays a critical role in the cellular antioxidant response. NRF2 is a basic leucine zipper protein (bZIP) transcription factor encoded by the *NFE2L2* gene that controls the expression of over 200 cytoprotective genes, including antioxidant proteins. Under normal conditions, NRF2 is kept inactive by Kelch‐like enoyl‐CoA hydratase‐associated protein 1 (KEAP1) and is degraded after its polyubiquitination by the Cullin 3 (Cul3) E3 ubiquitin ligase by the proteasome in the cytoplasm (Ngo and Duennwald 2022). However, when ROS accumulate and induce a stress response, NRF2 is released from KEAP1, which undergoes a conformational change after the modification of specific stress‐sensing cysteine residues. This results in NRF2 stabilization and its nuclear translocation. There, it binds to the antioxidant response element (ARE) in the promoter regions of genes encoding antioxidant proteins, such as heme oxygenase 1 (HO‐1) (Dai et al. 2020). Disruption of the NRF2 pathway in HGPS has been identified as a key driver of the premature aging phenotype. Kubben et al. (2016) demonstrated that progerin can sequester NRF2 at the nuclear lamina, hindering its transcriptional activity and exacerbating chronic oxidative stress.

MicroRNAs (miRNAs) are a class of endogenous small non‐coding RNAs (19–25 nucleotides) that regulate gene expression by targeting messenger RNAs (mRNAs). They primarily bind to the 3′ untranslated region (3′ UTR) of mRNAs, leading to either translational repression or mRNA degradation (Wahid et al. 2010). Dysregulation of miRNA expression has been implicated in a variety of human diseases, including cancer and neurodegenerative disorders. A number of miRNAs have been implicated in laminopathies and associated with deleterious effects (Frankel et al. 2018). While only a few miRNAs have been linked to HGPS pathophysiology, previous studies have shed light on their potential involvement in the disease. For instance, miR‐9, highly expressed in neurons, protects neural cells from progerin accumulation by targeting the 3′ UTR of wild‐type prelamin A and progerin transcripts, triggering their degradation (Jung et al. 2012; Nissan et al. 2012). Other miRNAs, all overexpressed in HGPS cells, have been described to contribute to HGPS pathogenesis, such as miR‐1 and miR‐29 (Ugalde et al. 2010, 2011), miR‐34a‐5p (Manakanatas et al. 2022), miR‐376a‐3p, and miR‐376b‐3p (Frankel et al. 2022) or miR‐59 (Hu et al. 2023).

To deepen our understanding of the role of miRNAs in HGPS, we conducted a comprehensive miRNA expression profiling using next‐generation sequencing (NGS) on dermal fibroblasts obtained from HGPS, HGPS‐like patients, and healthy controls. We demonstrated the critical role of miR‐140‐5p in HGPS fibroblasts as its overexpression represses NRF2, a key regulator of the antioxidant response. This repression contributes to increased oxidative stress, which could ultimately drive cellular senescence and participate in the premature aging phenotype characteristic of HGPS.

## 2 Results

### 2.1 Profiling of miRNA Expression Identifies miR‐140‐5p and miR‐140‐3p Overexpression in HGPS Fibroblasts

To identify miRNAs dysregulated in HGPS, we performed miRNA expression profiling using NGS on primary fibroblasts from 4 HGPS, 1 HGPS‐like, 2 age‐matched controls and a healthy 82‐year‐old donor for comparison with physiological aging. The HGPS cohort included four classical cases with the heterozygous *LMNA* c.1824C>T (p.G608G) mutation (HGPS1, HGPS2, HGPS3, and HGPS4) and one HGPS‐like patient (HGPS‐L) with the heterozygous *LMNA* c.1968 + 1G>A mutation (Table S1). This HGPS‐like patient, previously described by our team (Navarro et al. 2004), exhibited a severe neonatal premature aging syndrome and succumbed to respiratory distress at six months. Like the classical HGPS patients, HGPS‐L produces the prelamin AΔ50 transcript (progerin) (Barthélémy et al. 2015). Since miRNA expression in HGPS patients' fibroblasts is closely related to progerin accumulation, which increases with cell passage (Harhouri et al. 2017), we adjusted the cell passages used for our NGS analysis to account for progerin detection in patients' cells (Figure S1).

**FIGURE 1:**
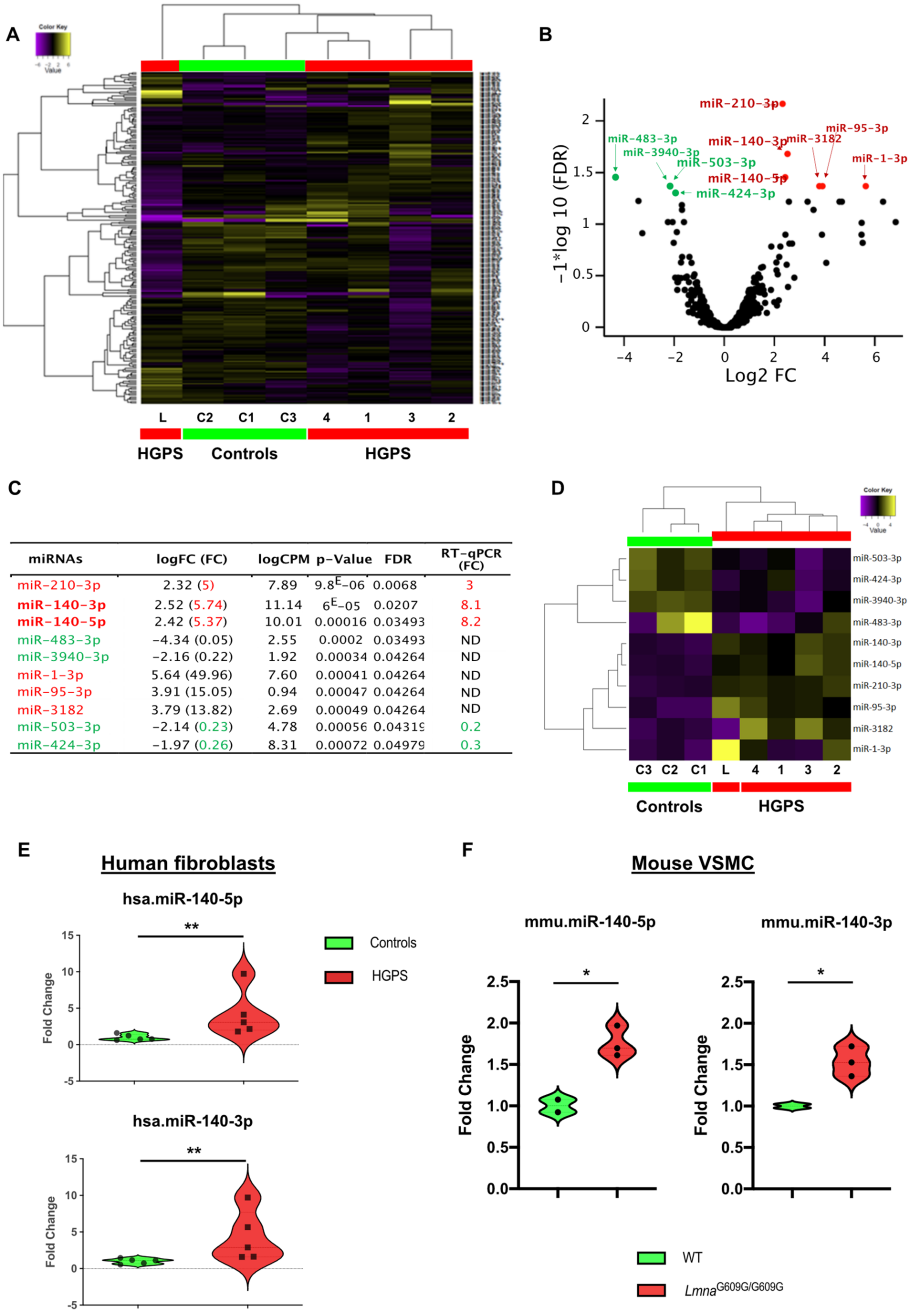
miRNA expression profile in HGPS fibroblasts. (A) Unsupervised hierarchical clustering of all expressed miRNAs in HGPS (red) and control (green) fibroblasts. Color intensity indicates miRNA expression level (purple = lower, yellow = higher). (B) Volcano plot showing the relationship between unadjusted *p*‐value (*y*‐axis, −log10 transformed) and fold change (*x*‐axis, log2 transformed) for miRNAs in HGPS/HGPS‐like versus controls. Green dots represent under‐expressed and red dots represent overexpressed miRNAs (*p* < 0.05, unpaired *t*‐test). (C) Table showing the 10 differentially expressed miRNAs in HGPS/HGPS‐like patients versus controls. LogFC (log fold change), fold change (FC), False discovery rate (FDR) correction for 693 comparisons, and counts per million (CPM) are shown. Under‐expressed miRNAs in patients are colored in green and overexpressed miRNAs are in red. Statistical results (*p*‐value and FDR) and individual RT‐qPCR validation results are included. ND, not determined. (D) Unsupervised hierarchical clustering of the 10 differentially expressed miRNAs in HGPS (red) and control (green) fibroblasts. Color intensity indicates miRNA expression level (purple = lower, yellow = higher). (E) Expression of hsa‐miR‐140‐5p and hsa‐miR‐140‐3p quantified by RT‐qPCR in HGPS fibroblasts (*n* = 5) as compared with the control fibroblasts (*n* = 5). Violin plots show the frequency distribution of the data. All individual data points are shown. **p < 0.01, Mann–Whitney test. (F) Expression of mmu‐miR‐140‐5p and mmu‐miR‐140‐3p quantified by RT‐qPCR in cultured VSMC from two aorta of *Lmna* ^G609G/G609G^ mice (*n* = 6) as compared with wild‐type mice (*n* = 4). Violin plots show the frequency distribution of the data. All individual data points are shown. *p ≤ 0.05, unpaired Welch's *t*‐test.

The miRNA‐Seq analysis identified 693 miRNAs. Unsupervised hierarchical clustering of all expressed miRNAs separated the samples into distinct subgroups (Figure 1A). Notably, the four HGPS patients (HGPS1, HGPS2, HGPS3, and HGPS4) clustered together with the elderly subject (C3, 82 years), while the other two controls (C1 and C2) formed a separate group. The HGPS‐L patient was clearly distinct from all others.

To identify miRNA of interest, we applied a selection pipeline (Figure S1). Statistical analyses revealed 10 differentially expressed (DE) miRNAs between the HGPS/HGPS‐L group and control cells (FDR < 0.05) among the 693 expressed miRNAs (Figure 1B,C). Among these 10 DE miRNAs, 6 were overexpressed and 4 were underexpressed (Figure 1C). Unsupervised hierarchical clustering of these 10 differentially expressed miRNAs clearly separated patients and controls (Figure 1D). Importantly, within the patient group, HGPS‐L was distinct from the 4 classical HGPS cases, while in the control group, the elderly subject (C3) was separated from the 2 younger controls.

To refine the selection of miRNAs of interest, we analyzed raw count plots and excluded miRNAs with low expression levels (less than 200 counts for both groups) or heterogeneous expression (presence of outliers in a group) (Figure S3A–C). This step eliminated 5 additional miRNAs. RT‐qPCR assays on mRNA extracts used for NGS confirmed the deregulation of the remaining 5 miRNAs (Figure 1C). Three miRNAs were overexpressed (miR‐210‐3p, miR‐140‐5p, and miR‐140‐3p), and two were underexpressed (miR‐503‐3p and miR‐424‐3p) in the HGPS/HGPS‐like group. Considering the numerous targets of miR‐140‐5p and miR‐140‐3p, which are involved in pathways known to be dysregulated in HGPS or to contribute to its phenotypic manifestations (Toury et al. 2022) (Figure S10), we prioritized these 2 miRNAs for further study.

To confirm the overexpression of miR‐140‐5p and miR‐140‐3p in a larger patient cohort and to assess their impact on cellular aging and senescence, we quantified each of these two miRNAs by RT‐qPCR in five patients (HGPS2, HGPS3, HGPS4, two new patients HGPS5, HGPS7) together with five controls (C1, C2, C3 and two new controls C4 and C5) at different passages. We confirmed the overexpression of miR‐140‐5p (FC = 4.18, *p* = 0.0079) and miR‐140‐3p (FC = 4.29, *p* = 0.0079) in HGPS fibroblasts compared to controls (Figure 1E). While the expression of miR‐140‐5p and miR‐140‐3p were highly correlated (*R* ^2^ = 0.9783, *p* < 0.001) (Figure S3D), there was no significant correlation between the expression of either miRNA and the level of senescence (*R* ^2^ = 0.1539, *p* = ns for miR‐140‐5p and R2 = 0.1769, *p* = ns for miR‐140‐3p) (Figure S1).

To evaluate the relevance of these 2 miRNAs in an animal model context, we further quantified the expression of mmu‐miR‐140‐5p and mmu‐miR‐140‐3p by RT‐qPCR in primary cultures of vascular smooth muscle cells (VSMCs) extracted from the thoracic aorta of *Lmna* ^G609G/G609G^ mice (*n* = 6) compared with wild‐type mice (*n* = 4). The aorta that exhibits a loss of VSMCs was shown to be severely affected in this progeria mouse model (Hamczyk et al. 2018). Similar to human HGPS fibroblasts, we observed overexpression of these 2 miRNAs in VSMCs from *Lmna* ^G609G/G609G^ mice (miR‐140‐5p, FC = 1.76, *p* = 0.0218 and miR‐140‐3p FC = 1.54, *p* = 0.0168) (Figure 1F).

### 2.2 HGPS Fibroblasts Exhibit a Downregulation of the NRF2/KEAP1/HO‐1 Antioxidant Pathway

Previous studies have shown that miR‐140‐5p can enhance oxidative stress by directly targeting NRF2 and SIRT2 mRNAs, thereby exacerbating atherosclerosis (Liu et al. 2019). Given our observation of miR‐140‐5p overexpression in HGPS/HGPS‐like patient fibroblasts, we investigated NRF2 expression under basal conditions in four distinct HGPS cell lines (HGPS1, HGPS2, HGPS3, and HGPS5) and two distinct controls (C2, C4).

**FIGURE 2:**
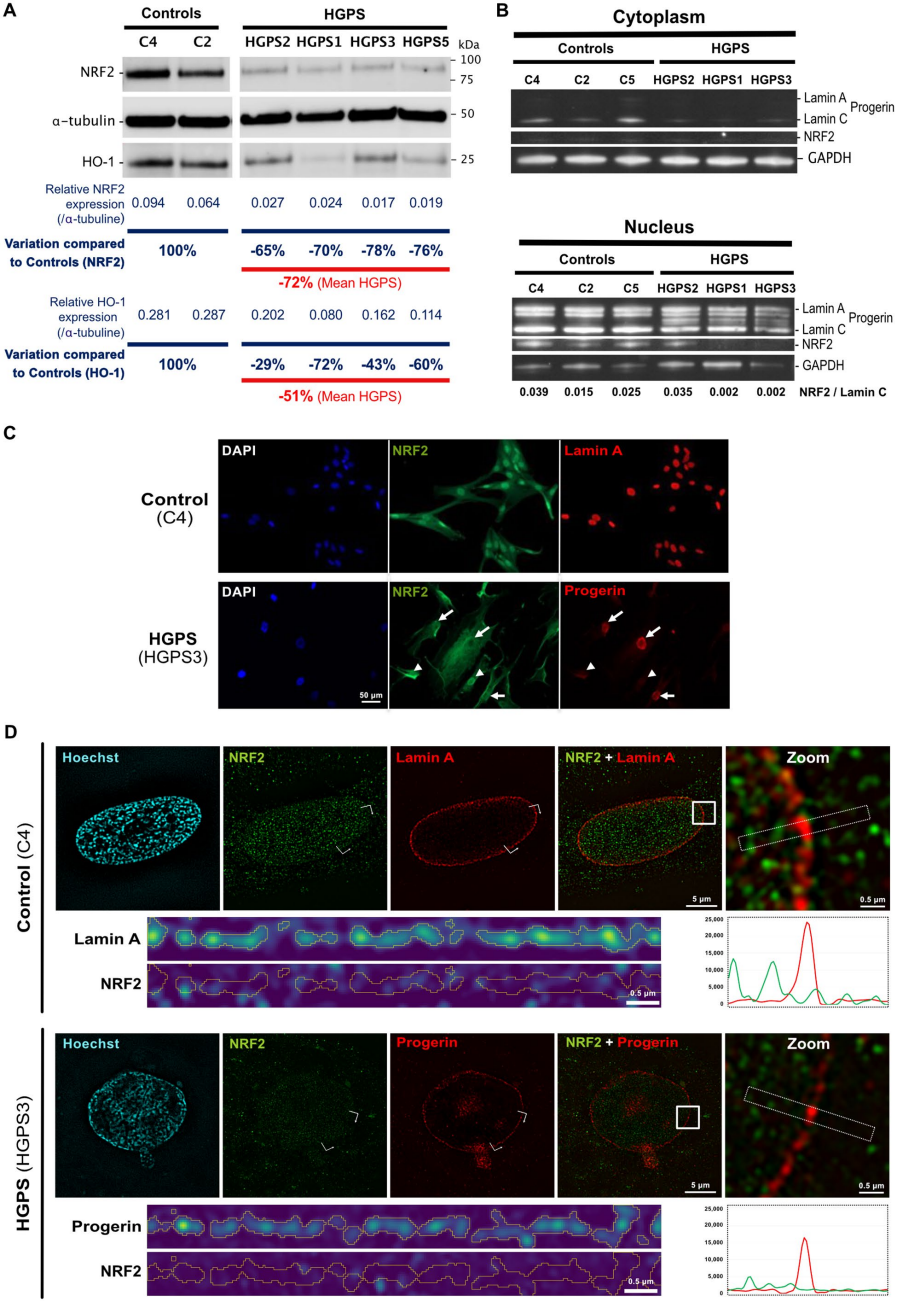
Downregulation of the NRF2/KEAP1/HO‐1 antioxidant pathway in HGPS fibroblasts. (A) Western blot analysis of whole‐cell lysates from controls and HGPS fibroblasts (2 cell lines for controls and 4 cell lines for HGPS). Detection of lamin A/C, progerin, NRF2, HO‐1, and α‐tubulin. Individual data points are shown. Relative expression, corresponding to the ratio of NRF2 or HO‐1 expression to α‐tubulin expression, are shown. Variation of NRF2 or HO‐1 expression compared to the mean expression of Controls is shown for each HGPS cell line (B) Western blot analysis of cytoplasmic and nuclear protein fractions from controls and HGPS fibroblasts (3 cell lines per group). Detection of lamin A/C, progerin, NRF2, and GAPDH. (C) Representative immunofluorescence images of control and HGPS fibroblasts stained for NRF2 and A (control) or progerin (HGPS) and counterstained with DAPI. Arrowheads indicate nuclei expressing NRF2 but not progerin. Arrows indicate nuclei expressing progerin but not NRF2. Scale bar = 50 μm. (D) Representative 3D‐SIM images of control (C4) and HGPS3 patient nuclei. Nuclei are labeled with NRF2 (green), Lamin A/Progerin (red), and counterstained with Hoechst (blue). Each image shows a single optical slice at the nuclear equator, extracted from a full *z*‐stack acquisition. The white square highlights the magnified region (Zoom), the dotted white rectangle indicates the area used for intensity profiling of Lamin A/Progerin and NRF2 (corresponding intensity profiles are displayed beneath the Zoom panels), and the white bracket marks the region of the straightened nuclear lamina (shown below the non‐zoomed images). All scale bars are indicated in the corresponding images. Full image acquisition and analysis procedures are described in the Supporting Information.

We found a reduced NRF2 protein level in all HGPS fibroblasts compared to controls (mean decrease: 72%) (Figure 2A). This decreased level is associated with a decrease in level of the antioxidant enzyme HO‐1, which is known to be transcriptionally regulated by NRF2, also observed in all HGPS fibroblasts (mean decrease: 51%) (Figure 2A). Additionally, we observed a significant reduction of KEAP1 level (Figure S4A). KEAP1 is the protein partner that sequesters NRF2 in the cytoplasm for degradation under normal conditions (Ngo and Duennwald 2022).

Using a fractionation protocol to isolate cytoplasmic and nuclear NRF2 protein fractions, we also observed differences between HGPS and controls. As expected, NRF2 was not detected in the cytoplasmic fraction of either controls or HGPS cells, as it is typically imported into the nucleus upon activation or alternatively degraded via the proteasome in conjunction with its partner KEAP1. We observed a dramatic decrease in nuclear NRF2 in HGPS1 and HGPS3 (99% and 86% compared to the mean expression in controls, respectively), while NRF2 was detectable in HGPS2 (Figure 2B).

At the transcriptional level, while NRF2 transcript *(NEF2L2)* level remained unchanged, HO‐1 transcript *(HMOX1)* level was significantly decreased in HGPS fibroblasts (*p* ≤ 0.05) (Figure S4B).

This heterogeneity regarding nuclear NRF2 distribution aligns with immunofluorescence staining observations showing that while NRF2 is consistently detected in all nuclei of control fibroblasts, the protein displays a heterogeneous pattern in HGPS fibroblasts (Figures 2C and S5). Interestingly, loss of nuclear NRF2 in HGPS fibroblasts is often associated with a high level of progerin accumulation, with some variability between cell lines. High‐resolution 3D structured illumination microscopy (SIM) confirmed these observations and further showed a marked reduction of nuclear NRF2 level in most cells with a high progerin level, without colocalization of the two proteins (Figures 2D and S6, Videos S1 and S2). Moreover, in rare nuclei displaying both high progerin and NRF2 levels, NRF2 is never enriched at the nuclear lamina and is not colocalized with progerin, excluding major sequestration of NRF2 by progerin (Figure S6).

These findings collectively suggest a defect in the NRF2/KEAP1/HO‐1 antioxidant pathway in HGPS fibroblasts, probably due to a loss of NRF2 expression that impairs its nuclear import.

### 2.3 Increased ROS Levels in HGPS Fibroblasts Correlates With Progerin Accumulation and NRF2/KEAP1 Pathway Repression

**FIGURE 3:**
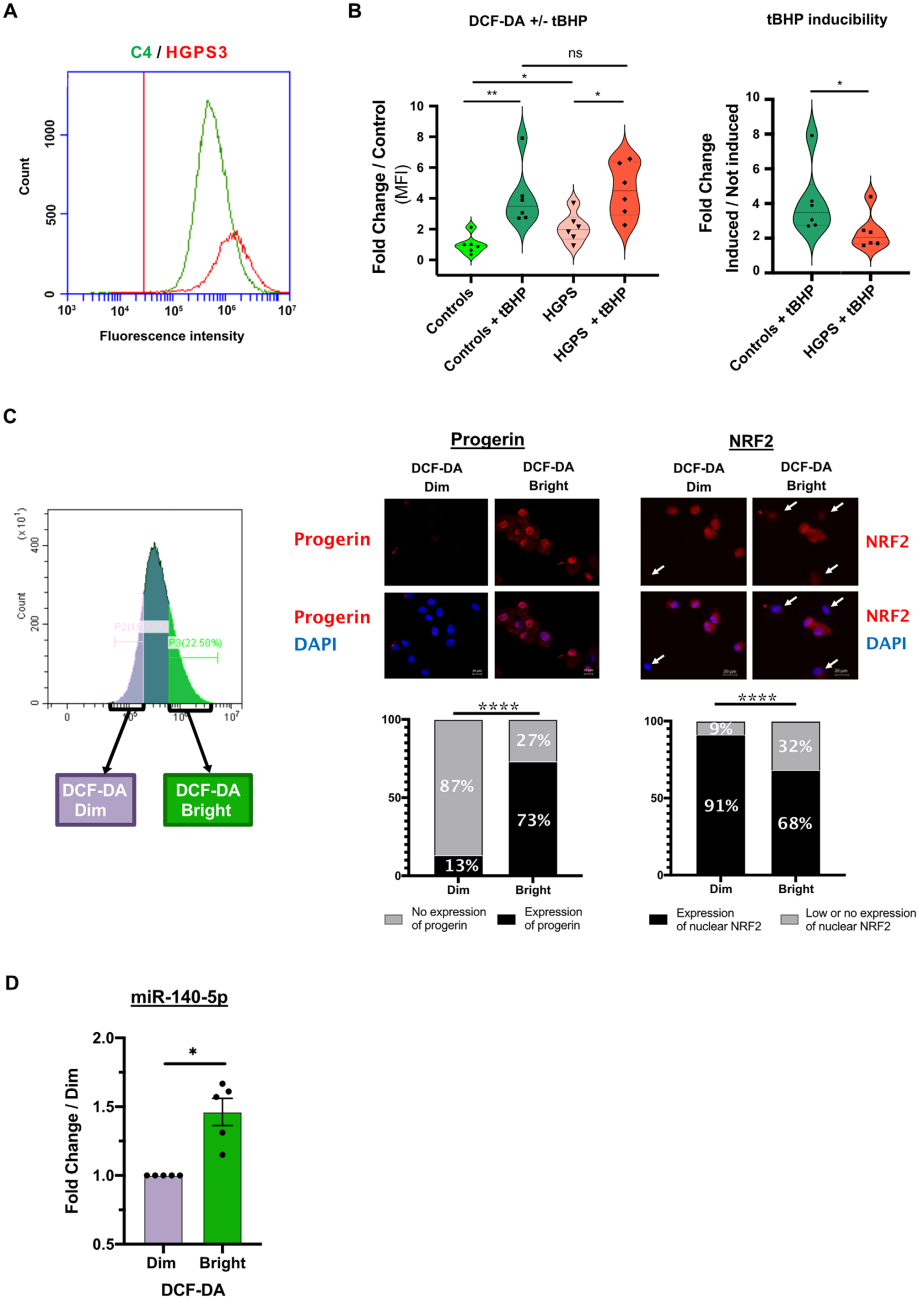
Increased ROS levels in HGPS fibroblasts resulting from NRF2/KEAP1 pathway repression. (A) Representative flow cytometry graph showing ROS levels in HGPS (HGPS3, red) and control (C4, green) fibroblasts using DCF‐DA. Positivity threshold was established using non stained cells. Histograms show cell count (*y*‐axis) and fluorescent intensity (*x*‐axis). (B) Statistical analysis comparing median fluorescent intensity (MFI). Violin plots show the distribution of MFI relative fold change for each condition (with or without tBHP‐induced oxidative stress) compared to control without tBHP. Right plots show the capacity of control and HGPS cells to respond to induced stress. All data points are shown. (ns, not significant; *p ≤ 0.05, **p < 0.01, Mann–Whitney test). (C) Representative flow cytometry graph showing two sorted cell populations (DCF‐DA Dim = low ROS, DCF‐DA Bright = high ROS) using FACS. Immunofluorescence images of sorted HGPS fibroblasts stained for progerin and counterstained with DAPI. Scale bar = 20 μm. Arrows indicate nuclei with low or no expression of NRF2. Graphs under images show percentages of cells with or without nuclear expression of progerin (left) and NRF2 (right). (****p < 0.0001, chi‐squared test). (D) Expression of miR‐140‐5p quantified by RT‐qPCR in low ROS fibroblasts (DCF‐DA Dim) as compared with high ROS fibroblasts (DCF‐DA Bright) (*n* = 5 for each). Values are expressed as fold change compared to DCF‐DA Dim normalized to SNORD38B. Plots show the frequency distribution of the data. All individual data points are shown (*p* = 0.0312, Wilcoxon test).

Given that the NRF2/KEAP1 pathway is a primary antioxidant system that mitigates excessive ROS production and oxidative stress, we quantified basal ROS levels by DCF‐DA by flow cytometry in HGPS (HGPS3) and control (C4) fibroblasts. We also assessed cellular responses to oxidative stress induced by tert‐Butyl Hydroperoxide (tBHP). As previously reported, HGPS fibroblasts exhibited higher basal ROS levels compared to controls (mean FC = 2.09, *p* = 0.0411) (Figure 3A,B). Both HGPS and control fibroblasts responded to tBHP induction (*p* = 0.0022 for control; and *p* = 0.0122 for HGPS), but the response magnitude was lower in HGPS cells compared to controls (mean FC = 2.37 and mean FC = 4.08, respectively, *p* = 0.0411) (Figure 3B).

In order to independently study two HGPS fibroblast subpopulations exhibiting low (DCF‐DA Dim) or high (DCF‐DA Bright) ROS content, we sorted them by Fluorescence‐Activated Cell Sorting (FACS), according to DCF‐DA levels (Figure 3C). We observed a positive association between ROS levels and progerin expression, as cells with high ROS levels (DCF‐DA Bright) had a higher proportion of progerin‐positive cells (73%) compared to cells with low ROS levels (DCF‐DA Dim) (13%) (*p* < 0.0001) (Figure 3C). In contrast, a negative association was observed between ROS levels and nuclear NRF2 level, as cells with high ROS levels (DCF‐DA Bright) had a higher proportion of nuclear NRF2‐negative cells (32%) compared to cells with low ROS levels (DCF‐DA Dim) (9%) (*p* < 0.0001). Quantification of miR‐140‐5p in each of the two HGPS fibroblast populations depending on ROS levels revealed a higher expression of miR‐140‐5p in DCF‐DA Bright compared to DCF‐DA Dim population (*n* = 5, mean FC = 1.462 ± 0.099, *p* = 0.0312) (Figure 3D). Notably, a significantly higher expression of miR‐140‐3p was also observed in the DCF‐DA Bright population compared to the DCF‐DA Dim population (*n* = 5, mean FC = 1.391 ± 0.081, *p* = 0.0312) (data not shown).

These data suggest that the defective antioxidant NRF2 pathway in HGPS fibroblasts, likely associated with the presence of progerin, contributes to ROS accumulation.

### 2.4 miR‐140‐5p Overexpression Represses the NRF2/KEAP1/HO‐1 Pathway, Leading to ROS Accumulation in HGPS Fibroblasts

**FIGURE 4:**
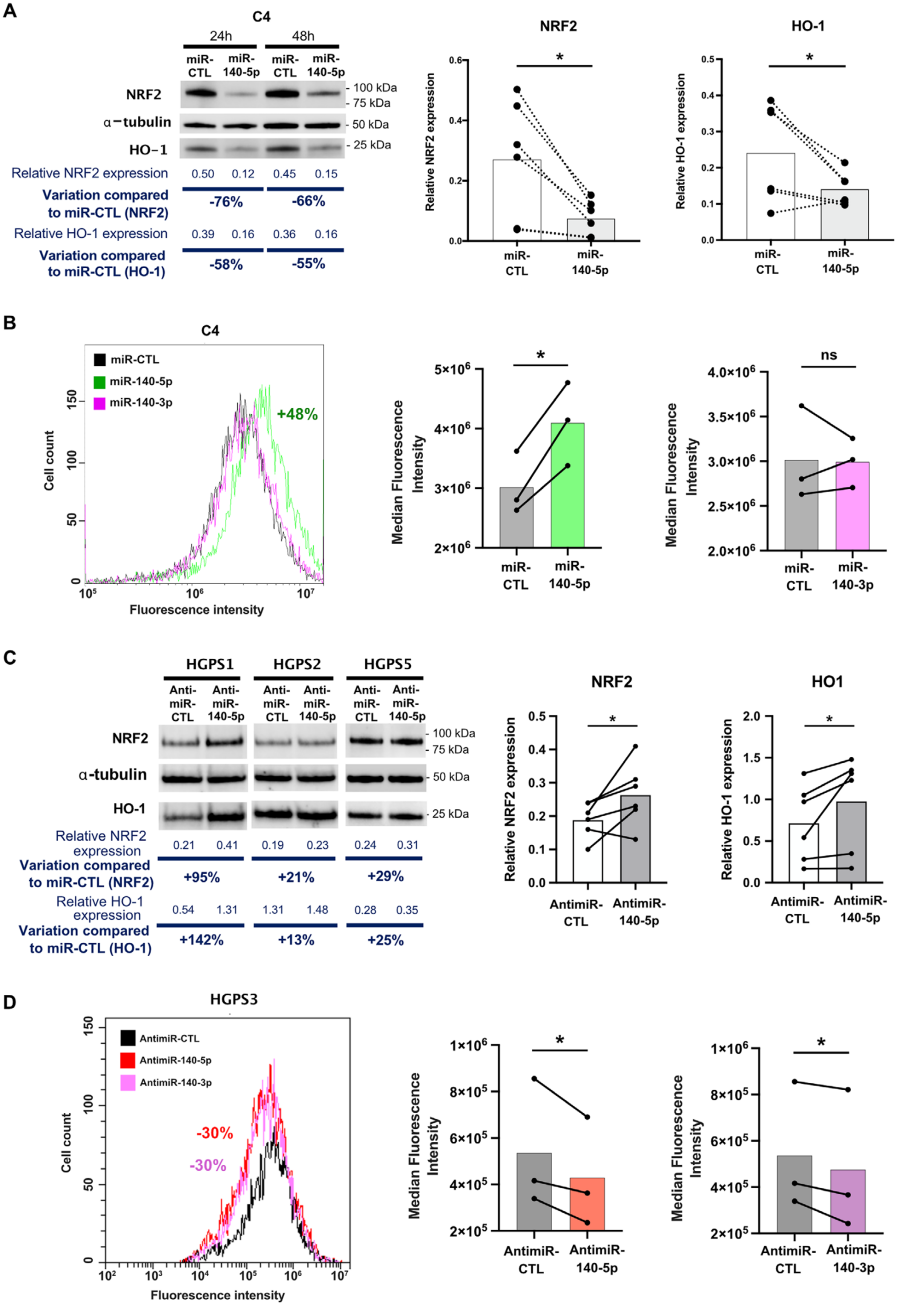
miR‐140‐5p overexpression represses the NRF2/KEAP1/HO‐1 pathway. (A) Representative Western blot of whole‐cell lysates from control (C4) fibroblasts after miR‐140‐5p transfection compared to miR‐control (miR‐CTL) at 24 and 48 h. Immunodetection of NRF2, HO‐1, and α‐tubulin. Relative expression, corresponding to the ratio of NRF2 or HO‐1 expression to α‐tubulin expression, is shown. The first graph at right summarizes relative NRF2 protein levels for each condition (miR‐140‐5p or miR‐CTL) at 24 and 48 h post transfection, with the average indicated by the bars (Wilcoxon test, *n* = 6, *p ≤ 0.05). The second graph at right represents relative HO‐1 protein levels with or without miR‐140‐5p transfection, with the average indicated by the bars (one‐tailed Wilcoxon test, *n* = 6, *p ≤ 0.05). (B) Representative flow cytometry graph showing the effect of miR‐140‐5p, miR‐140‐3p, or miR‐CTL transfection on ROS levels in control (C4) fibroblasts, measured using DCF‐DA. Histograms show cell count (*y*‐axis) and fluorescence intensity (*x*‐axis). The graph on the right compares the median fluorescence intensity (MFI) for each condition. Violin plots show the distribution of MFI across samples (paired *t*‐test, ns, not significant; *p ≤ 0.05, *n* = 3 per condition). (C) Western blot analysis shows NRF2, HO‐1, and α‐tubulin protein levels in HGPS (HGPS1, HGPS2, and HGPS5) fibroblasts after transfection with antimiR‐140‐5p (inhibits miR‐140‐5p) or antimiR‐control (antimiR‐CTL) at 48 h. The graphs summarize relative protein levels (NRF2 and HO‐1) for each condition (antimiR‐140‐5p or antimiR‐CTL), with the average indicated by the bars (*n* = 3 cell lines with 2 times post‐transfection, Wilcoxon test). (D) Representative flow cytometry graph showing the effect of antimiR‐140‐5p, antimiR‐140‐3p, or antimiR‐CTL transfection on ROS levels in HGPS (HGPS3) fibroblasts, measured using DCF‐DA. Histograms show cell count (*y*‐axis) and fluorescence intensity (*x*‐axis). The graph on the right compares the median fluorescence intensity (MFI) for each condition. Violin plots show the distribution of MFI across samples (*p ≤ 0.05, paired *t*‐test, one‐tailed, *n* = 3 per condition).

To investigate the role of miR‐140‐5p overexpression in ROS accumulation through NRF2/KEAP1/HO‐1 pathway repression, we transfected miR‐140‐5p mimic (miR‐140‐5p) and a control mimic (miR‐CTL) into three different control fibroblast cell lines (C2, C4, and C6). We observed a significant decrease in NRF2 protein level after miR‐140‐5p transfection (*p* = 0.0312) with a similar effect 48 h post‐transfection (73% mean decrease; 66% decrease for C4) compared to 24 h (70% mean decrease; 76% decrease for C4) (Figures 4A and S7A). As expected, this decrease in NRF2 is associated with a decrease in HO‐1 (*p* = 0.0312) at both time points (58% decrease at 24 h and 55% decrease at 48 h for C4) (Figure 4A).

To confirm the impact of miR‐140‐5p‐induced NRF2/KEAP1/HO‐1 pathway inhibition on ROS accumulation, we assessed ROS levels by flow cytometry following transfection of miR‐140‐5p, miR‐CTL, and miR‐140‐3p (which does not target NRF2 mRNA). As expected, miR‐140‐5p overexpression in control fibroblasts (C4) significantly increased ROS accumulation (mean = +36%, *p* = 0.0252), while miR‐140‐3p had no effect (mean = −0.3%, *p* = 0.9008) (Figure 4B).

Next, we transfected antimiR‐140‐5p and antimiR control (antimiR‐CTL) into 3 different HGPS cell lines (HGPS1, HGPS2 and HGPS5). As expected, antimiR‐140‐5p transfection increased NRF2 and HO‐1 expression, although the timing and magnitude of the effect varied between the 3 cell lines (NRF2: mean = +44.5%, *p* = 0.0312; HO‐1: mean = +40.5%, *p* = 0.0312) (Figure 4C). In HGPS1, antimiR‐140‐5p transfection led to a high response for NRF2 (+120%) and a weaker response for HO‐1 (+27%) as early as 24 h post‐transfection whereas we observed a marked effect on HO‐1 level 48 h post‐transfection (+95% for NRF2 and +142% for HO‐1) (Figure S7B). In HGPS2 and HGPS5, the effect of antimiR‐140‐5p is delayed and weaker than in HGPS1 (NRF2: +21% and HO‐1: +13% for HGPS2; NRF2: +29% for and HO‐1: +25% for HGPS5, 48 h post‐transfection). At 72 h post‐transfection, the effect of antimiR‐140‐5p remained stable for HGP2 (+21% for NRF2 and +29% for HO‐1) but was lost for HGPS5 (−19% for NRF2 and +6% for HO‐1) (Figure S7B). Finally, the inhibition of miR‐140‐5p in HGPS fibroblasts (HGPS3) led to a significant decrease in ROS accumulation 48 h post‐transfection (mean = −21% compared to antimiR‐CTL, *p* = 0.0404) (Figure 4D). Notably, the inhibition of miR‐140‐3p in HGPS fibroblasts (HGPS3) also led to a significant decrease (mean = −15% compared to antimiR‐CTL, p = 0.0420) of ROS accumulation at 48 h post‐transfection (Figure 4D).

These results demonstrate that miR‐140‐5p overexpression in HGPS fibroblasts leads to the inhibition of the NRF2/KEAP1/HO‐1 pathway, resulting in ROS accumulation that contributes to chronic oxidative stress.

### 2.5 miR‐140‐5p Overexpression Leads to Mitochondrial Dysfunction in HGPS Fibroblasts

Previous studies described mitochondrial dysfunction in HGPS fibroblasts (Mateos et al. 2015; Xiong et al. 2016; Kang et al. 2017). To investigate the contribution of miR‐140‐5p to this dysfunction, we quantified the oxidative phosphorylation (OXPHOS) mitochondrial function by analyzing the oxygen consumption rate (OCR) which reflects mitochondrial respiration using a Seahorse assay. Simultaneously, the extracellular acidification rate (ECAR) that corresponds to protons excretion and reflects glycolysis, was measured.

**FIGURE 5:**
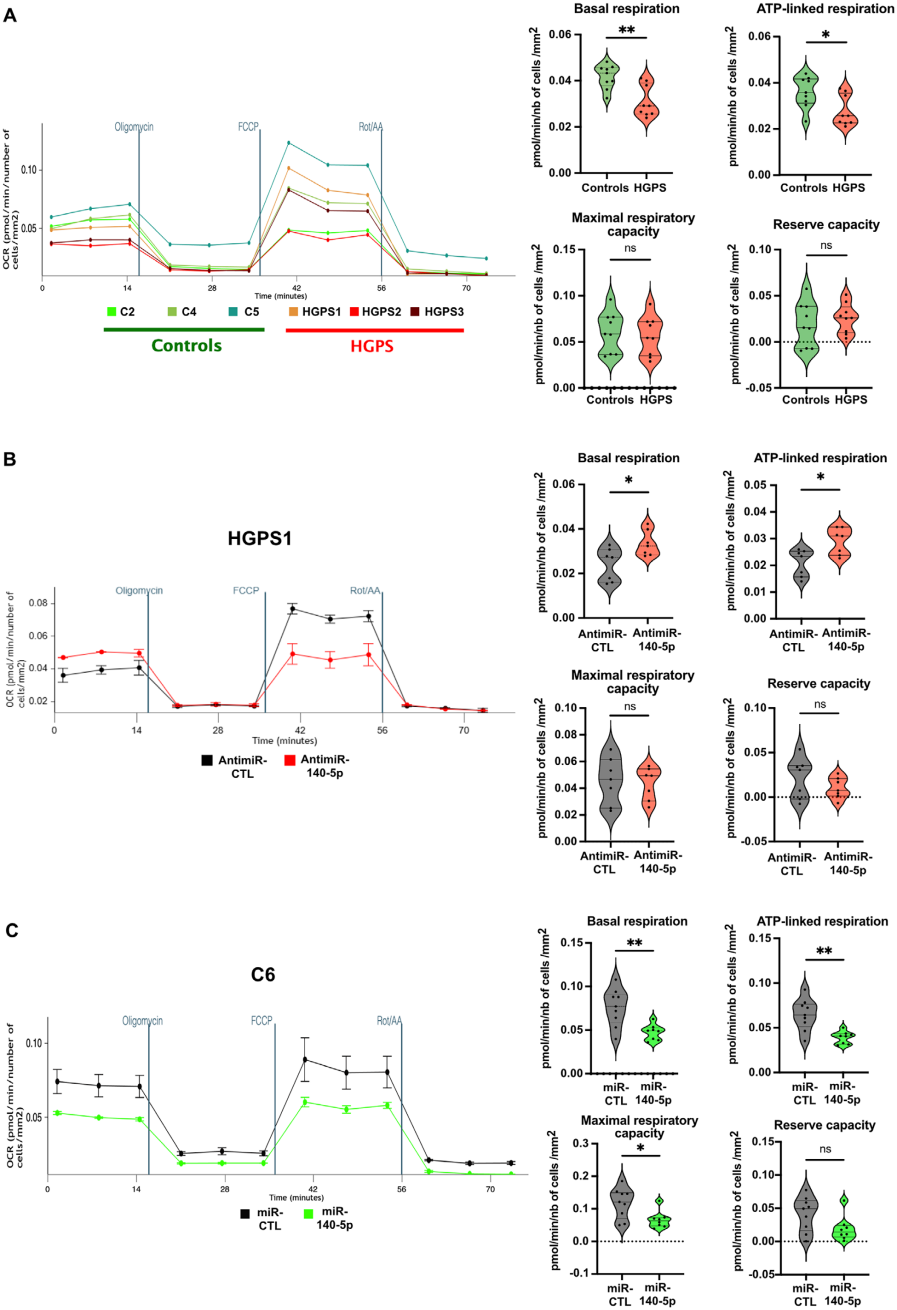
miR‐140‐5p overexpression leads to mitochondrial dysfunction in HGPS fibroblasts. (A) Seahorse assay comparing OCR levels between control (C2, C4, and C5) and HGPS (HGPS1, HGPS2, and HGPS3) fibroblasts. The graphs on the right compare basal respiration, ATP‐linked respiration, maximal respiratory capacity, and reserve capacity. Violin plots show the distribution of OCR across samples (unpaired *t*‐test and Mann–Whitney test, ns, not significant; *p ≤ 0.05, **p < 0.01, 3 repeated OCR were measured per condition). (B) Representative Seahorse assay showing the effect of antimiR‐140‐5p transfection at 48 h on OCR measurement in HGPS fibroblasts (HGPS1). The graphs on the right compare basal respiration, ATP‐linked respiration, maximal respiratory capacity, and reserve capacity. Violin plots show the distribution of OCR across samples (Mann–Whitney test, ns, not significant; *p ≤ 0.05, *n* = 3 cell lines (HGPS1, HGPS3, and HGPS5), *n* = 2 or 3 experiments per condition). (C) Representative Seahorse assay showing the effect of miR‐140‐5p transfection at 48 h on OCR measurement in control fibroblasts (C6). The graphs on the right compare basal respiration, ATP‐linked respiration, maximal respiratory capacity, and reserve capacity. Violin plots show the distribution of OCR across samples (unpaired *t*‐test or Mann–Whitney test, ns, not significant; *p ≤ 0.05, **p < 0.01, *n* = 3 experiments per condition).

We first analyzed OCR and ECAR in control and HGPS fibroblasts. Interestingly, basal respiration and ATP‐linked respiration were significantly altered in HGPS cells (*p* = 0.0056 and 0.0296, respectively) (Figure 5A). In parallel, glycolytic reserve capacity was significantly enhanced in HGPS cells (*p* = 0.0039) (Figure S8A). In contrast, no significant differences were observed in maximal respiratory capacity, reserve capacity, and basal ECAR (*p* = 0.547, 0.6048, and 0.837) (Figures 5A and S8A).

We then transfected antimiR‐140‐5p and an antimiR control (antimiR‐CTL) into three different HGPS cell lines (HGPS1, HGPS3, and HGPS5). Inhibition of miR‐140‐5p overexpression led to improvement of basal and ATP‐linked respiration (*p* = 0.0262 and *p* = 0.0379, respectively) (Figure 5B), whereas no effect was observed on maximal respiratory capacity, reserve capacity, basal ECAR, and glycolytic reserve capacity (*p* > 0.999, 0.3829, 0.9015, and 0.5350, respectively) (Figures 5B and S8B).

The mirror experiment was carried out by ectopically overexpressing miR‐140‐5p in control fibroblasts. Overexpression of miR‐140‐5p in control cells (C6) mimicked, at least in part, the phenotype observed in HGPS cells by inducing a clear reduction of basal respiration and ATP‐linked respiration (*p* = 0.003 and 0.0015, respectively) (Figures 5C and S8C). This overexpression had also a negative effect on maximal respiratory capacity and glycolytic reserve capacity (*p* = 0.0360 and *p* = 0.206, respectively), while no effect was observed on the reserve capacity and basal ECAR.

Our results demonstrate that basal and ATP‐linked respiration are altered in HGPS cells and suggest that, in our model, this compromised OXPHOS mitochondrial function may be compensated by increased glycolysis reserve capacity. Moreover, we demonstrate that overexpression of miR‐140‐5p contributes to these mitochondrial alterations.

### 2.6 miR‐140‐5p Overexpression Affects Other Hallmarks of Aging in HGPS Fibroblasts

Beside mitochondrial dysfunction, HGPS fibroblasts classically display other hallmarks of aging and several characteristic defects, including nuclear dysmorphias, reduced proliferation, impaired DNA repair, and cellular senescence (Cau et al. 2014).

**FIGURE 6:**
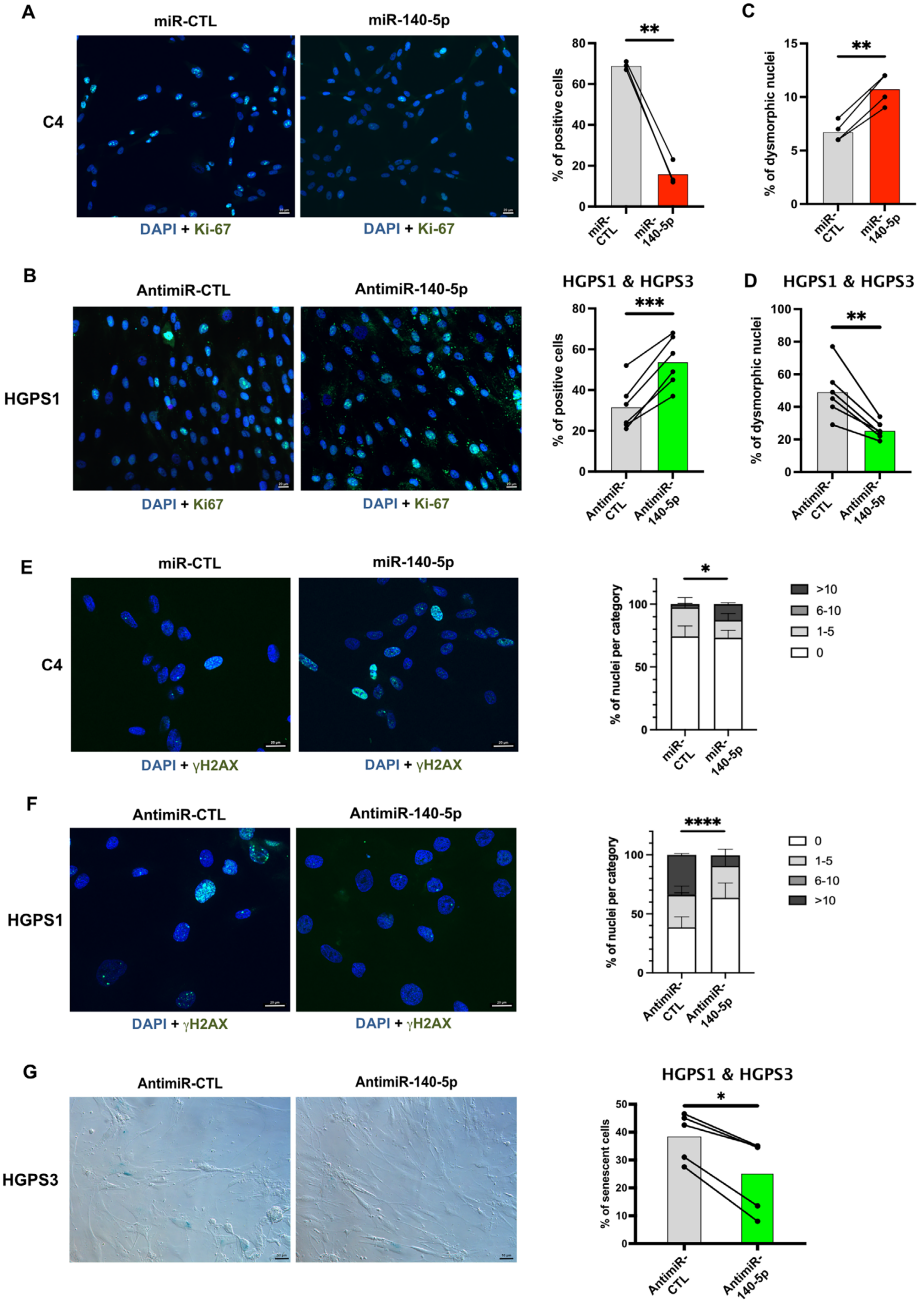
miR‐140‐5p overexpression affects several hallmarks of aging in HGPS fibroblasts. (A, B) Representative immunofluorescence images of Ki‐67 (green). Nuclei were counterstained with DAPI (blue). Scale bar = 20 μm. Right: Quantification of Ki‐67‐positive cells (%). Hundred nuclei were counted per condition. Bars represent mean values. (A) Control fibroblasts (C4) transfected with miR‐140‐5p or miR‐control (miR‐CTL) for 48 h (paired *t*‐test, *n* = 3; **p = 0.0025). (B) HGPS fibroblasts (HGPS1) transfected with antimiR‐140‐5p or antimiR‐control (CTL) for 48 h. Quantification of Ki‐67‐positive cells in HGPS1 and HGPS3 (paired *t*‐test, *n* = 3 per line; ***p = 0.0003). (C, D) Quantification of nuclear abnormalities. Hundred nuclei were counted per condition. The percentage of dysmorphic nuclei was plotted. (C) Control fibroblasts (C4) transfected with miR‐140‐5p or miR‐CTL for 48 h (paired *t*‐test, *n* = 4; **p = 0.0023). (D) HGPS fibroblasts (HGPS1 and HGPS3) transfected with antimiR‐140‐5p or antimiR‐CTL for 48 h (paired *t*‐test, *n* = 3 per line; **p = 0.0037). (E, F) Representative immunofluorescence images of γH2AX (green) for quantification of DNA damages. Nuclei were counterstained with DAPI (blue). Scale bar = 20 μm. Right: Quantification of the number of foci of γH2AX cell, classified into categories (0; 1–5; 6–10; > 10). Hundred nuclei were counted per condition. (E) Control fibroblasts (C4) transfected with miR‐140‐5p or miR‐CTL for 48 h (Fisher's exact test, *n* = 3; *p = 0.0137). (F) HGPS fibroblasts (HGPS1) transfected with antimiR‐140‐5p or antimiR‐CTL for 48 h (Fisher's exact test, *n* = 3; ****p < 0.0001). (G) Colorimetric detection of senescence‐associated β‐galactosidase in HGPS fibroblasts (HGPS1) transfected with antimiR‐140‐5p or antimiR‐CTL for 48 h. Scale bar = 50 μm. Right: Quantification of senescent cells (%) in HGPS1 and HGPS3. Hundred nuclei were counted per condition. Bars represent mean values (Wilcoxon, one‐tailed, *n* = 3 for HGPS1 and 2 for HGPS3; *p = 0.0312).

We first investigated the effect of miR‐140‐5p on cell proliferation. Overexpression of miR‐140‐5p in control cells (C4) markedly reduced the Ki‐67 proliferation marker compared to miR‐CTL (mean = −76.8%, *p* = 0.0025) (Figure 6A). Conversely, inhibition of miR‐140‐5p with antimiR‐140‐5p in two HGPS cell lines (HGPS1 and HGPS3) significantly increased Ki‐67 levels (mean = +70%, *p* = 0.0003) (Figures 6B and S9A). These findings were further confirmed in two control cell lines (C4 and C5) using complementary proliferation assays. Both the CellTiter‐Glo Luminescent Cell Viability Assay (ATP‐based quantification of viable cells) and the BrdU incorporation assay (DNA synthesis during S phase) consistently showed reduced proliferation upon miR‐140‐5p overexpression (Figure S9B). Specifically, transfection of miR‐140‐5p mimics into control fibroblasts (C4 and C6) significantly decreased luminescence in the CellTiter‐Glo assay (*p* = 0.0003), without detectable cytotoxicity in the CellTox assay (*p* = 0.1953), indicating reduced proliferation rather than increased cell death. Similarly, BrdU incorporation was significantly reduced in miR‐140‐5p–transfected cells (*p* = 0.0119), suggesting impaired proliferation and potential cell cycle arrest. Together, these results indicate that miR‐140‐5p negatively regulates fibroblast proliferation.

We next examined nuclear architecture. Overexpression of miR‐140‐5p in control fibroblasts (C4) increased nuclear dysmorphias (mean = +59.3%, *p* = 0.0023), but the percentage remained low (10.75%). Conversely, antimiR‐140‐5p transfection in HGPS1 and HGPS3 cells improved nuclear morphology (mean = −48.1%, *p* = 0.0037) (Figure 6C,D). These results suggest that miR‐140‐5p contributes, directly or indirectly, to the nuclear architecture defects.

To evaluate DNA repair capacity, we quantified DNA damages using γH2AX staining. Overexpression of miR‐140‐5p in control fibroblasts (C4) significantly increased γH2AX foci (*p* = 0.0137) (Figure 6E), while anti‐miR‐140‐5p reduced γH2AX foci in HGPS1 (*p* < 0.0001) and HGPS3 (*p* = 0.0359) (Figures 6F and S9C), supporting a role for miR‐140‐5p in promoting DNA damage accumulation.

Finally, we assessed cellular senescence using colorimetric detection of senescence‐associated β‐galactosidase. Transfection of antimiR‐140‐5p in HGPS1 and HGPS3 fibroblasts modestly reduced senescence levels (mean = −34.5%, *p* = 0.0312) (Figure 6G and S9D), whereas overexpression of miR‐140‐5p in control fibroblasts (C4) did not significantly affect senescence (*p* = 0.250). This limited effect may be due to the relatively short duration of exposure (48 h post‐transfection), insufficient to trigger major senescence changes.

In summary, our results demonstrate that miR‐140‐5p overexpression in HGPS fibroblasts contributes not only to mitochondrial dysfunction but also to several additional hallmarks of aging, including impaired proliferation, nuclear dysmorphias, defective DNA repair, and increased cellular senescence.

## 3 Discussion

In this study, we investigated the role of miRNAs in the pathogenesis of Hutchinson‐Gilford Progeria Syndrome, a rare premature aging disorder caused by mutations in the *LMNA* gene. Our miRNA profiling analysis identified 10 significant DE miRNAs in HGPS fibroblasts compared to healthy controls. Among them, we identified the two overexpressed miRNAs miR‐140‐5p and miR‐140‐3p, produced from the same precursor, as good candidates based on their ability to target pathways described to be altered in HGPS and/or associated with the clinical phenotype (Figure S10).

In this study, we focused on miR‐140‐5p, which has been implicated in various cellular processes relevant to HGPS, including oxidative stress, cell proliferation, senescence, and skeletal development (Toury et al. 2022). Notably, miR‐140‐5p directly targets the mRNAs of NRF2 and SIRT2, two key regulators of the antioxidant defense system, thereby increasing oxidative stress (Liu et al. 2019), which is a hallmark of aging (López‐Otín et al. 2023), and a central factor in the HGPS pathogenesis (Cau et al. 2014). The critical role of the NRF2 pathway in HGPS pathophysiology has been previously highlighted by Kubben et al. (2016). They demonstrated that progerin impairs NRF2 transcriptional activity by sequestering it at the nuclear lamina, thereby preventing the activation of the antioxidant response element. Our study provides new insights into the molecular regulation of the NRF2 pathway in HGPS. Indeed, we observed a downregulation of both NRF2 and its cytoplasmic partner KEAP1, leading to a decreased NRF2 activity in HGPS fibroblasts. We show that miR‐140‐5p overexpression in HGPS fibroblasts represses the NRF2/KEAP1/HO‐1 antioxidant pathway, thus contributing to the disease phenotype. The repression of NRF2 by miR‐140‐5p occurs at the translational level as the transcript level of NRF2 *(NFE2L2)* is not decreased. Protein fractionation and immunofluorescence analyses confirmed reduced nuclear NRF2 levels in any, but not all, HGPS cell lines. Consequently, the expression of the NRF2‐regulated antioxidant enzyme, HO‐1, was significantly decreased at the transcript and protein levels, despite elevated cellular ROS levels. The observed downregulation of KEAP1 in HGPS fibroblasts may be a secondary consequence of reduced cellular NRF2 levels induced by miR‐140‐5p overexpression, potentially leading to KEAP1 degradation. In addition to its direct regulation of *NFE2L2*, miR‐140‐5p may also destabilize NRF2 indirectly by simultaneously targeting both Pin1 and the PI3K/AKT pathway. Surprisingly, a pro‐oxidative effect of miR‐140‐3p was also observed in our model. As miR‐140‐3p does not directly target NRF2 transcript, other regulatory mechanisms may be proposed. SIRT1 (Sirtuin 1) and GSTM3 (Glutathione S‐transferase mu 3), both key actors in preventing oxidative stress, are proven or potential targets of miR‐140‐3p, respectively.

Our data also demonstrate the negative impact of miR‐140‐5p overexpression on mitochondrial function by affecting OXPHOS in HGPS fibroblasts. Mitochondrial dysfunction is a well‐established feature of HGPS, characterized by aberrant morphology, reduced mobility, decreased ATP production, and impaired mitogenesis due to decreased peroxisome proliferator‐activated receptor‐gamma coactivator‐1 alpha (PGC‐1α) expression (Mateos et al. 2015; Xiong et al. 2016; Kang et al. 2017). This dysfunction is partially attributed to progerin‐mediated ROS accumulation (Arnold et al. 2021). Our findings suggest that miR‐140‐5p contributes to this mitochondrial dysfunction, but the molecular mechanisms may be multifactorial. One potential mechanism involves the downregulation of NRF2, which is also a key regulator of mitochondrial function. NRF2 plays a crucial role in maintaining mitochondrial membrane potential, regulating substrate availability for respiration, and supporting mitochondrial biogenesis and integrity during inflammation and oxidative stress (Vomund et al. 2017).

The NRF2 decrease induced by miR‐140‐5p overexpression could significantly contribute to HGPS pathophysiology. As demonstrated by Kubben et al. (2016), NRF2 knockdown in wild‐type fibroblasts or mesenchymal stem cells derived from genetically corrected HGPS iPSCs (GC‐iPSC‐MSCs) mimics several HGPS defects even if the toxic progerin protein is absent, including increased ROS levels and reduced antioxidant gene expression. Notably, NRF2 knockdown in GC‐iPSC‐MSCs also resulted in increased stem cell attrition, mirroring the observed stem cell dysfunction in HGPS. Conversely, experimental elevation of NRF2 levels in HGPS cells restored NRF2 pathway activity and improved some HGPS phenotypes. In vivo studies further support the role of NRF2 in HGPS. *C. elegans* SKN‐1 mutants (NRF2 homolog) display a shortened lifespan (An and Blackwell 2003). Nrf2‐deficient mice also exhibit a shortened lifespan (Yoh et al. 2001), reduced bone mineral density (Ibáñez et al. 2014), and other aging‐related phenotypes, such as decreased adipogenic differentiation and altered lipid profiles (Tanaka et al. 2008), which are also observed in HGPS. These findings, along with the observed effects of NRF2 knockdown in vitro, strongly suggest that NRF2 dysregulation plays a significant role in the pathogenesis of HGPS.

In our study, we did not observe sequestration of NRF2 at the nuclear *lamina* as previously described by Kubben et al. (2016). High‐resolution microscopy failed to reveal colocalization of NRF2 and progerin aggregates at the nuclear periphery in the two HGPS fibroblast lines analyzed and instead confirmed a marked reduction of nuclear NRF2 compared with controls. This discrepancy might be attributed to the use of different cellular models. Our work focused exclusively on primary fibroblast cultures from HGPS patients and did not rely on induced progerin overexpression. Notably, we observed substantial heterogeneity among HGPS cell lines, both in the extent of progerin accumulation and in the degree of NRF2 downregulation. Furthermore, the repression of the NRF2/KEAP1/HO‐1 pathway varied between patients and across culture passages. Interestingly, miR‐140‐5p overexpression was detected at early passages, independent of the degree of senescence. Preliminary data further suggest that miR‐140‐5p upregulation may represent a common hallmark of progeroid syndromes, not directly dependent on progerin accumulation, and potentially linked to epigenetic modifications at chromosome 16q22 miR‐140 gene locus (data not shown). Although we cannot exclude that progerin may contribute to NRF2 sequestration as described previously (Kubben et al. 2016), our data indicate that miR‐140‐5p‐mediated NRF2 repression constitutes a major mechanism amplifying the overall downregulation of the NRF2 pathway in HGPS fibroblasts.

MicroRNAs (miRNAs) are potent regulators of gene expression, capable of simultaneously targeting numerous mRNAs due to their small size. Beyond NRF2, miR‐140‐5p targets other key molecules relevant to HGPS pathophysiology. Firstly, miR‐140‐5p targets Peptidyl‐prolyl isomerase 1 (Pin1), an enzyme crucial for various cellular processes including cell cycle regulation and growth. Pin1 deregulation contributes to aging, cancer, and neurodegenerative diseases (Li et al. 2021, 1). Notably, Pin1 is essential for bone cell differentiation, regulating key signaling pathways like Wnt, BMP (bone morphogenetic protein), and FGF (fibroblast growth factor) (Islam et al. 2017). Furthermore, Pin1 downregulation promotes senescence in tendon stem/progenitor cells (Chen et al. 2015, 1). Secondly, miR‐140‐5p targets Transforming Growth Factor‐β receptor I (TGFBR1), a key component of the TGF‐β and BMP signaling pathways crucial for skeletal development and maintenance (Wu et al. 2016; Zhang et al. 2015). TGF‐β signaling inhibits adipogenesis and promotes osteoblast differentiation, and TGFBR1 knockdown enhances adipogenesis and reduces osteoblast differentiation (Zhang et al. 2015). Finally, miR‐140‐5p targets transcripts of DNPEP (Aspartyl Aminopeptidase), BMP2 (Bone morphogenetic protein 2), and HDAC4 (Histone deacetylase 4), all critical regulators of chondroblastic and osteoblastic differentiation (Nakamura et al. 2011). In zebrafish embryos, microinjection of miR‐140‐5p disrupts bone development, inducing craniofacial and skeletal malformations by targeting BMP‐2 (Gan et al. 2016).

The diverse functions of miR‐140‐5p target genes, encompassing roles in oxidative stress, cellular senescence, adipogenesis, and skeletal development, suggest that its overexpression in HGPS cells may contribute significantly to the multifaceted aging phenotype observed in these patients. This includes characteristic features such as adipose tissue alterations (lipoatrophy, lipodystrophy) and various skeletal abnormalities (growth retardation, low bone density, osteoporosis, osteolysis, clavicular resorption, acro‐osteolysis, or arthritis). Therapeutic interventions aimed at modulating miR‐140‐5p could potentially address multiple dysfunctional molecular pathways contributing to the HGPS phenotype. As discussed by Kubben et al. (2016), restoring NRF2 activity may offer a novel therapeutic strategy for HGPS, given their identification of NRF2 pathway repression as a key driver of the disease. Moreover, our results show that miR‐140‐5p overexpression impacts more features and hallmarks of aging such as cell proliferation decrease, nuclear architecture loss, DNA repair defects (genomic instability), and cellular senescence (López‐Otín et al. 2023). Notably, modulating miR‐140‐5p overexpression could not only restore NRF2 expression and alleviate oxidative stress and its associated consequences but also potentially improve other aspects of the HGPS phenotype, including osteoarticular and cardiovascular diseases, by restoring the expression of multiple miR‐140‐5p target genes involved in these processes.

## 4 Conclusion

This study provides novel insights into the molecular mechanisms underlying Hutchinson‐Gilford Progeria Syndrome. Our findings demonstrate that miR‐140‐5p, upregulated in HGPS fibroblasts, contributes to the disease phenotype by repressing the NRF2 antioxidant pathway. By targeting NRF2 transcript, miR‐140‐5p exacerbates oxidative stress, a hallmark of HGPS. Furthermore, given the diverse functions of miR‐140‐5p target genes, including those involved in skeletal development, adipogenesis, and cellular senescence, its overexpression likely contributes to the multifaceted aging phenotype observed in HGPS patients. These findings highlight the potential of modulating miR‐140‐5p expression as a novel therapeutic strategy for HGPS, not only by restoring NRF2 activity and mitigating oxidative stress but also by potentially addressing other aspects of the disease, such as skeletal abnormalities and adipose tissue dysfunction.

## 5 Material and Methods

### 5.1 Cells

Dermal fibroblasts from HGPS patients and controls were sourced from skin biopsies, cultured, and stored either at the TAC Biological Resource Center (La Timone Hospital, Assistance Publique des Hôpitaux de Marseille, France) or obtained from the Coriell Institute for Medical Research and ATCC. All biological samples from the CRB TAC included signed informed consent for research use. In the manuscript, HGPS and control fibroblasts are referred to as detailed in Table S1. Culture conditions are described in Supporting Information.

### 5.2 Mice

Knock‐in mouse model *Lmna* ^G609G/G609G^ carrying the c.1827C>T (p.Gly609Gly) mutation (Osorio et al. 2011) and wild‐type mice were used for the study. Animal experiments have been carried out in compliance with the ARRIVE (Animal Research: Reporting of in vivo Experiments) guidelines and the European guidelines for the care and use of laboratory animals (EU directive 2010/63/EU). All animal procedures were carried out under protocols approved by a local and national ethical committee for animal experimentation (Ministère de l'Education Nationale, de l'Enseignement Supérieur et de la Recherche; Authorization Apafis N°7404‐2016102816469761 v4). Isolation and primary culture of mouse VSMCs were prepared as previously described (Cardoso et al. 2024) (see Supporting Information).

### 5.3 miRNA Expression Analysis by Next Generation Sequencing

Total RNA extractions, quality controls and miRNA sequencing have been sub‐contracted to Integragen (Evry, Paris, France) (see Supporting Information). Data has been processed by statistical analysis comparing the control group (*n* = 3) to the HGPS group (*n* = 5).

### 5.4 Quantification of Individual miRNAs by Quantitative RT‐PCR

cDNA was synthesized from extract RNA at a concentration of 5 ng/μL using the miRCURY LNA RT kit (Qiagen, Hilden, Germany), following the manufacturer's instructions. Expression levels of specific miRNAs were measured with the miRCURY LNA SYBR Green PCR Kit (Qiagen, Hilden, Germany). Quantitative PCR amplifications were carried out in triplicate using the miRCURY LNA miRNA PCR Assay primers for *hsa*‐miR‐140‐5p, *hsa*‐miR‐140‐3p, *mmu*‐miR‐140‐5p, and *mmu*‐miR‐140‐3p (Qiagen, Hilden, Germany) on a QuantStudio 5 Real‐Time PCR System (Thermo Fisher Scientific, Waltham, MA, USA). The threshold cycle (Cq) was used to calculate relative miRNA expression using the 2^−ΔΔCT^ method, normalized to *hsa*‐SNORD49A or *hsa*‐SNORD38B expression for human miRNAs or *mmu*‐SNORD110 expression for mouse miRNAs (Qiagen, Hilden, Germany).

### 5.5 Quantification of Individual mRNAs by Quantitative RT‐PCR

The extracted RNA was reverse transcribed using the SuperScript IV Reverse Transcriptase Kit (Invitrogen, Thermo Fisher Scientific, Waltham, MA, USA). The cDNA was amplified with specific primers for 40 cycles at a hybridization temperature of 59°C. Real‐time PCR amplification was conducted using the TaqMan Gene Expression Master Mix (Applied Biosystems) on a QuantStudio 5 Real‐Time PCR System (Thermo Fisher Scientific, Waltham, MA, USA) with predesigned primers for *NFE2L2* (Hs00975961_g1), *HMOX1* (Hs01110250_m1), *GAPDH* (Hs00266705_g1) and *RPS13* (Hs01011487_g1) (Thermo Fisher Scientific Scientific). All PCRs were performed in triplicate. Threshold cycle (Ct) values were used to determine relative mRNA expression using the 2^−∆∆CT^ method, normalized to GAPDH or RPS13 expression.

### 5.6 Transfection and Treatment

Mimics (miRIDIAN microRNA Mimic: hsa‐miR‐140‐5p, hsa‐miR‐140‐3p, Mimic Negative Control) were purchased from Horizon Discovery LTD (Cambridge, UK). miRNA inhibitors (‘antimiR’) (miRCURY LNA miRNA Power Inhibitors hsa‐miR‐140‐5p, has‐miR‐140‐3p miRCURY LNA microRNA power inhibitor control) were purchased from Qiagen (Hilden, Germany). Mimics (50 nM) and antimiR (25 nM) were transfected using a Lipofectamine RNAiMAX kit (Thermo Fisher Scientific, Waltham, MA, USA) according to the manufacturer's instructions. Endogenous overexpression of miR‐140‐5p in HGPS fibroblasts was confirmed by RT‐qPCR before antimiR transfection.

### 5.7 Senescence Associated β‐Galactosidase Staining

Senescence was assessed using the Senescence β‐Galactosidase Staining Kit (Cell Signaling Technology, Leiden, The Netherlands) following the manufacturer's protocol. The percentage of stained cells was determined under a microscope (Leica, Wetzlar, Germany) by two independent observers through manual blind counting. For each condition, at least 100 fibroblasts were randomly selected. The results are presented graphically as the mean percentage of senescent cells.

### 5.8 Western Blot Analysis

Equal amounts of protein (40 μg) were reduced at 95°C for 5 min with NuPAGE Reducing Agent (NP0009, Thermo Fisher Scientific, Waltham, MA, USA) and loaded into 8% Bis‐Tris gels (NuPAGE precast gel, Thermo Fisher Scientific, Waltham, MA, USA) using NuPAGE MES SDS Running Buffer (NP0002, Thermo Fisher Scientific, Waltham, MA, USA). After electrophoresis, proteins were transferred to Immobilon‐FL PVDF membranes (IPFL00010, Millipore, Burlington, MA, USA).

The primary antibodies used were: anti‐lamin A/C (1:2000, 10298–1‐AP, Proteintech, Manchester, UK), anti‐Progerin (1/50, sc‐81611, Santa Cruz Biotechnology, CA, USA), anti‐NRF2 (1:2000, 80593–1‐RR, Proteintech, Manchester, UK), anti‐HO‐1 (1:1000, 66743–1‐Ig, Proteintech, Manchester, UK), anti‐KEAP1 (1:2000, 10503–2‐AP, Proteintech, Manchester, UK), anti‐α‐tubulin (1:4000, T6199, Sigma Aldrich), and anti‐GAPDH (1:40,000, MAB374, Millipore, Burlington, MA, USA).

For revelation, ECL or fluorescence detection was used depending on the protein detected (see Supporting Information).

All blots were imaged using a ChemiDoc MP Imaging System (Bio‐Rad), and bands were quantified using Image Lab Software (Bio‐Rad, Hercules, CA, USA). GAPDH or α‐tubulin was used as a total cellular protein loading control.

### 5.9 Indirect Immunofluorescence

Fibroblasts were seeded into 4‐chamber well slides (SPL Lifesciences, Korea). Cells were fixed in 4% paraformaldehyde (PFA) for 15 min at room temperature, then permeabilized with 0.5% Triton X‐100 permeabilization buffer for 10 min at room temperature. Subsequently, cells were incubated with primary antibodies overnight at 4°C: anti‐progerin (1:50, sc‐81,611), anti‐lamin A (1:100, sc‐56,137, Santa Cruz Biotechnology, CA, USA), anti‐NRF2 (1:100, GTX103322, GeneTex, Irvine, CA, USA), anti‐Ki67 Rabbit (1/100, ab15580, Abcam, Cambridge, UK) and γH2AX (1/500, #2577, Cell Signaling Technology, Danvers, USA). After washing, cells were incubated with secondary antibodies (A21203 Donkey anti‐mouse AF594; A32754 Donkey anti‐Rabbit AF488, 1:500, Life Technologies) for 1 h at room temperature. Nuclei were stained with DAPI, and slides were mounted with Vectashield Antifade Mounting Medium (H‐1000‐10, Vector Laboratories, Newark, CA, USA), then observed using a fluorescence microscope (ApoTome.2, Zeiss, Oberkochen, Germany). After FACS based on DCF‐DA intensity, sorted stained cells were analyzed using IMARIS (Oxford Instruments, Abingdon‐on‐Thames, UK). Nuclei were selected and then separated into positive or negative nuclei according to progerin or NRF2 intensity (see Figure S11 for step‐by‐step process). For 3D Structured Illumination Microscopy (3D‐SIM), cells were cultured on high‐precision glass coverslips (No. 1.5H; 0117580, Marienfeld, Germany). Immunofluorescence staining was performed as described above. Secondary antibodies used were Donkey anti‐Rabbit AF488 (A11008, 1:500, Life Technologies) and Donkey anti‐mouse AF647 (A32787, 1:500, Life Technologies). Coverslips were mounted in SlowFade Glass Soft‐set Antifade Mountant (Thermo Fisher Scientific, #S36917). Data were obtained on a commercial N‐SIM system (Nikon) and analyzed as described in Supporting Information.

### 5.10 ROS Quantification

Intracellular ROS production was detected using the non‐fluorescent compound DCF‐DA (2′, 7′‐dichlorofluorescin diacetate). As it crosses plasmic membrane, the compound is deacetylated by intracellular esterases to form the non‐fluorescent DCF, which oxidizes to a highly fluorescent compound. For the DCF‐DA assay, cells were washed with PBS 1X (Thermo Fisher Scientific, Waltham, MA, USA) and incubated for 30 min at 37°C in Dulbecco's modified Eagle's medium low glucose, pyruvate, no glutamine, no phenol red (Thermo Fisher Scientific, Waltham, MA, USA) with 10 μM DCF‐DA (D6883‐50MG, Sigma Aldrich, St Louis, MO, USA). For positive controls of ROS production, 100 μM of Tert‐Butyl hydroperoxide (tBHP) (Sigma Aldrich, St Louis, MO, USA) was added to the medium. The cells were then washed with PBS 1X and detached from the culture flask using trypsin (Invitrogen, Thermo Fisher Scientific, Waltham, MA, USA) for 5 min and centrifuged at 250 *g* for 5 min. The supernatant was removed, and the pellet was washed twice with PBS 1X (Invitrogen, Thermo Fisher Scientific, Waltham, MA, USA). The cells were resuspended in 500 μL PBS 1X and their fluorescence spectra were determined using a Cytoflex S (Beckman Coulter Life Sciences, Brea, CA, USA) or BD Accuri C6 PLUS (BD Biosciences, Le Pont de Claix, France).

### 5.11 Fluorescence‐Activated Cell Sorting (FACS)

Cells were sorted using Cytoflex SRT (Beckman Coulter Life Sciences, Brea, CA, USA) after DCF‐DA experiment, according to DCF‐DA positivity. Two populations of cells were selected according to the level of ROS they contained, low (“dim” population) or high (“bright” population). Percentage of selected cells, number of cells and median fluorescence intensity were respectively 20.7% [range: 12.8–37.1]; 89,056 cells [range: 4753–289,447], and 215,630 [range: 74,478–514,589] for the “dim” population and 27.5% [range: 22.4–38.1]; 130,700 cells [range: 7494–414,438] and 1,114,255 [range: 509,468–3,001,244] for the “bright” population. The cells were subsequently prepared for immunofluorescence analysis or processed in QIAzol for RNA quantification.

### 5.12 Seahorse Assay

The oxygen consumption rate (OCR) and extracellular acidification rate (ECAR) were measured using the Agilent Seahorse XF Analyzer (Seahorse Bioscience Inc., North Billerica, MA, USA), and the Cell Mito Stress Test Kit (Agilent Technologies, Santa Clara, CA, USA), following the manufacturer's protocol and as previously described (Mishra et al. 2019). Cells were seeded at a density of 10,000 cells per well. OCR and ECAR measurements were taken as cells were sequentially treated with 1.5 μM oligomycin, a complex V inhibitor, 2 μM carbonyl cyanide‐p‐trifluoromethoxyphenylhydrazone (FCCP), a protonophore which collapses the inner membrane gradient, and 0.5 μM rotenone/antimycin A (Rot/AA), inhibitors of complex III and I, leading to shut down electron transport chain (ETC) function, revealing the non‐mitochondrial respiration.

OCR and ECAR were normalized to cell count. Basal respiration, ATP‐linked respiration, maximal respiratory capacity and reserve capacity were calculated as described in Supporting Information.

### 5.13 Quantification and Statistical Analysis

Statistical analysis was conducted using GraphPad Prism 8 (GraphPad Software Inc., San Diego, CA, USA). Normality tests were applied on the data to assess distributions, and we used the parametric or non‐parametric test appropriate for our data. The test used and the number of replicates are detailed in the corresponding figure legend. A *p*‐value ≤ 0.05 was considered significant, with significance levels indicated as follows: *p ≤ 0.05, **p < 0.01, ***p < 0.001, and ****p < 0.0001.

## Author Contributions

Conceptualization and supervision: Patrice Roll and Elise Kaspi. Experimentation: Léa Toury, Diane Frankel, Sara Nael, Léa Le Goff, Marie Basset, Coraline Airault, and Elise Kaspi. Data analyze: Léa Toury, Diane Frankel, Sara Nael, Mario Abaji, Léa Le Goff, Marie Basset, Bertrand Vernay, Elva María Novoa‐del‐Toro, Anaïs Baudot, Elise Kaspi, and Patrice Roll. Methodology: Patrice Roll, Elise Kaspi, Diane Frankel, and Frederique Magdinier. Writing: Léa Toury, Diane Frankel, Elise Kaspi, and Patrice Roll.

## Conflicts of Interest

The authors declare no conflicts of interest.

## Supporting information


**Appendix S1:** acel70276‐sup‐0001‐AppendixS1.

## Data Availability

All data supporting this study are included in the article and its Supporting Information files; additional datasets are available from the corresponding author upon request.
