# Supplementary material for: miR‐140‐5p Overexpression Contributes to Oxidative Stress and Mitochondrial Dysfunction in Hutchinson‐Gilford Progeria Syndrome Fibroblasts Through NRF2 Pathway
Source: Aging Cell. 2025 Oct 31;24(12):e70276. doi: 10.1111/acel.70276 (PMC12686586; doi:10.1111/acel.70276)
Supplement: Supplementary file 1 — Appendix S1: acel70276‐sup‐0001‐AppendixS1. [file ACEL-24-e70276-s001.zip › acel70276-sup-0001-AppendixS1/acel70276-sup-0002-Table S1.pdf]

	Patients								Controls							
**Phenotype**	**HGPS**							**HGPS-like**	**Wild-type**							
**ID**	**HGPS1**	**HGPS2**	**HGPS3**	**HGPS4**	**HGPS5**	**HGPS6**	**HGPS7**	**HGPS-L**	**C1**	**C2**	**C3**	**C4**	**C5**	**C6**	**C7**	**C8**
**Corresponding name**	13-8243	13-5968	AG 11498	AG 01972	AG 11513	AG 03199	13-13622	13-15288	HFF-1	AG 08498	13-10742	AG 07095	13-13090	AG 05966	GM 08398	AG 08471
***LMNA* genotype**	c.1824C>T p.G608G	c.1824C>T p.G608G	c.1824C>T p.G608G	c.1824C>T p.G608G	c.1824C>T p.G608G	c.1824C>T p.G608G	c.1824C>T p.G608G	c.1968+1 G>A	WT	WT	WT	WT	WT	WT	WT	WT
**miRNA-Seq** (passage)	Yes *(P10)*	Yes *(P13)*	Yes *(P17)*	Yes *(P20)*	No	No	No	Yes *(P13)*	Yes *(P12)*	Yes *(P17)*	Yes *(P7)*	No	No	No	No	No
**Age at collection (years)**	2	5	14	14	8	10	2	6	< 1	1	82	2	7	12	8	NA
**Sex**	Female	Female	Male	Female	Female	Female	Male	Female	Male	Male	Female	Male	Male	Male	Male	Male
**Source**	CRB	CRB	Coriell	Coriell	Coriell	Coriell	Coriell	CRB	ATCC	Coriell	CRB	Coriell	CRB	Coriell	Coriell	Coriell

WT: wild-type

Table S1: Identification and source of primary fibroblasts. This table provides details about the origin of the fibroblast cells used in the study. All HGPS patients carry a specific mutation in the *LMNA* gene (c.1824C>T, p.Gly608Gly), while the HGPS-like patient has a different mutation (c.1968+1G>A) that also leads to progerin production. RefSeq: NM_170707.4. *Homo sapiens* lamin A/C *(LMNA)*, transcript variant 1, mRNA. Coriell: Coriell Institute for Medical Research *(https://www.coriell.org/)*. NA: not available.
