# Supplementary material for: miR‐140‐5p Overexpression Contributes to Oxidative Stress and Mitochondrial Dysfunction in Hutchinson‐Gilford Progeria Syndrome Fibroblasts Through NRF2 Pathway
Source: Aging Cell. 2025 Oct 31;24(12):e70276. doi: 10.1111/acel.70276 (PMC12686586; doi:10.1111/acel.70276)
Supplement: Supplementary file 1 — Appendix S1: acel70276‐sup‐0001‐AppendixS1. [file ACEL-24-e70276-s001.zip › acel70276-sup-0001-AppendixS1/acel70276-sup-0001-Supplemental Methods.pdf]

Supplemental Methods

Culture of human fibroblasts

Fibroblasts were cultured in Dulbecco's modified Eagle's medium low glucose (VWR, Radnor, Pennsylvania, USA) with the addition of 15% fetal bovine serum (FBS) (Thermo Fisher Scientific, Waltham, MA, USA), 2 mM L-glutamine (Thermo Fisher Scientific, Waltham, MA, USA), and a mixture of 100 U/mL penicillin, streptomycin (Thermo Fisher Scientific, Waltham, MA, USA), at 37°C in a humidified atmosphere with 5% CO_2. For western blot experiments to study NRF2 pathway, fibroblasts were incubated with 10 µM MG132 for 6 hours before protein extraction. Fibroblast pellets selected for miRNA expression studies were stored at −80 °C until analyses.

Primary culture of mouse VSMCs

Knock-in mouse model *Lmna*$^{G609G/G609G}$ carrying the c.1827C>T (p.Gly609Gly) mutation (Osorio et al., 2011) and wild-type mice were used for the study.
Isolation and primary culture of mouse VSMCs were prepared as previously described (Cardoso et al. 2024). Briefly, VSMCs were extracted from thoracic aorta of one-month *Lmna*$^{G609G/G609G}$ mice (n=6) and wild-type mice (n=4). To have enough VSMC to start the culture, 2 aortas from 2 mice of the same littermate were pooled into the HBSS in 15 ml conical in a cool place. After incubation at 37°C for 8-10 minutes with enzyme solution containing collagenase, soybean trypsin inhibitor, elastase, 1% penicillin/streptomycin and HBSS, aortas were washed with DMEM/F12 (Gibco) media before removing endothelial cell layer. Aortas were opened longitudinally to remove any blood clots and endothelial cell layer and incubated at 37°C in 5% CO_2 for about an hour within enzyme solution. After digestion, the digested tissue was triturate by shredding, wash with DMEM/F12 media and centrifuge 3 times. After centrifugation, VSMCs were cultured on 2 wells of a 24 wells dish DMEM/F12 media supplemented with FBS (10%) and penicillin/streptomycin (1%). Primary VSMC pellets of each cell line were prepared at passage 8 were stored at −80 °C until miRNA expression studies.

RNA extraction for quantitative RT-PCR

Total RNA was extracted from dermal fibroblasts or murine VSMCs using the miRNeasy kit (Qiagen, Hilden, Germany) according to the manufacturer’s instructions. As low amounts of RNAs were expected after cells selection by FACS, MS2 RNA bacteriophage (Sigma) was added as an RNA carrier in order to avoid miRNAs loss during RNA isolation (Ramón-Núñez et al. 2017).
Samples were quantified by absorbance using a NanoDrop DN-1000 spectrophotometer (Thermo Fisher Scientific, Waltham, MA, USA).

Protein extraction and quantification for Western blot analysis

Total fibroblast proteins were extracted using a lysis buffer containing 62.5 mM Tris-HCl, 2.3% SDS, 10% glycerol, and 1 mM PMSF, followed by sonication three times for 30 seconds each.
Nuclear and cytoplasmic fractions of proteins were obtained using NE-PER Nuclear and Cytoplasmic Extraction Kit according to the manufacturer protocol (Thermo Fisher Scientific, Waltham, MA, USA). After collection of each fraction, extracts were rapidly store at -80°C until use.
Protein concentration was determined using the Pierce™ BCA Protein Assay Kit according to the manufacturer's instructions (Thermo Fisher Scientific, Waltham, MA, USA). Absorbance was measured with a UV-Visible Absorbance Module (540–590 nm bandpass) on a GloMax® Microplate Reader (Promega, Madison, WI, USA).

miRNA Expression Analysis by Next Generation Sequencing

Our previous studies have shown that miRNA expression is strongly influenced by progerin accumulation in HGPS cells and that, depending on the HGPS cell line, the number of passages does not clearly reflect the level of accumulation (Frankel et al. 2022). For this reason, we selected, for each HGPS cell line, a passage in which over 90% of the cells expressed progerin quantified by immunofluorescence analysis, and with progerin expression confirmed by western blot **(Figure S1)**. Total RNA extractions, quality controls and miRNA sequencing have been sub-contracted to Integragen (Evry, Paris, France). Briefly, cell pellets containing 4 to 5 million fibroblasts were shipped to Integragen on dry ice. Total RNAs were extracted using the miRNeasy mini kit (Qiagen, Hilden, Germany) according to the manufacturer's instructions. RNA integrity was assessed using an Agilent 2100 Bioanalyzer (Agilent Technology, Santa Clara, CA, USA). All samples showed a high quality and integrity (RNA integrity number (RIN) > 7) and were selected for NGS. Library was next prepared for sequencing. NGS was performed on Illumina HiSeq™4000, with one flow cell per pooled library and with a read length of 42 base pairs per read, including a 6 base pair index sequence, and a data yield of approximately 8-12 million reads per sample. The raw sequencing data were processed to remove adapter sequences and demultiplexed to separate individual samples. Raw reads were checked for potential sequencing issues and contaminants using FastQC. The clean reads were aligned against the mature miRNA in miRBase20.0 (http://www.mirbase.org/) to identify known miRNAs. The read counts of each known miRNA were then normalized to the total counts of sequence reads mapped to the miRBase version 20.0 database to obtain reads per million mapped reads (RPM). This data has been processed by statistical analysis comparing the control group (n=3) to the HGPS group (n=5).

Western blot analysis

Total fibroblast proteins were extracted using a lysis buffer containing 62.5 mM Tris-HCl, 2.3% SDS, 10% glycerol, and 1 mM PMSF, followed by sonication three times for 30 seconds each.

Nuclear and cytoplasmic fractions of proteins were obtained using NE-PER Nuclear and Cytoplasmic Extraction Kit according to the manufacturer protocol (Thermo Fisher Scientific, Waltham, MA, USA). After collection of each fraction, extracts were rapidly store at -80°C until use.
Protein concentration was determined using the Pierce™ BCA Protein Assay Kit according to the manufacturer's instructions (Thermo Fisher Scientific, Waltham, MA, USA). Absorbance was measured with a UV-Visible Absorbance Module (540–590 nm bandpass) on a GloMax® Microplate Reader (Promega, Madison, WI, USA).
Equal amounts of protein (40 µg) were reduced at 95°C for 5 minutes with NuPAGE Reducing Agent (NP0009, Thermo Fisher Scientific, Waltham, MA, USA) and loaded into 8% Bis-Tris gels (NuPAGE™ precast gel, Thermo Fisher Scientific, Waltham, MA, USA) using NuPAGE™ MES SDS Running Buffer (NP0002, Thermo Fisher Scientific, Waltham, MA, USA). After electrophoresis, proteins were transferred to Immobilon-FL PVDF membranes (IPFL00010, Millipore, Burlington, MA, USA).
ECL revelation: Membranes were blocked in 5% nonfat dry milk diluted 1:1 in PBS for 1 hour at room temperature, then incubated overnight at 4°C with primary antibodies diluted in TBS (50 mM Tris, 150 mM NaCl, pH 7.6) with 0.1% Tween 20 (Bio-Rad) (TBS-T buffer).
Blots were washed with TBS-T buffer and incubated with secondary antibodies: anti-Mouse IgG HRP-linked Antibody (7076S, Cell Signaling Technology, Leiden, The Netherlands) or anti-Rabbit IgG HRP-linked Antibody (7074S, Cell Signaling Technology, Leiden, The Netherlands) at 1:3,000 dilution and Precision Protein™ StrepTactin-HRP Conjugate at 1:10,000 dilution in TBS-T buffer for 1 hour at room temperature.
Fluorescence revelation: Membranes were blocked for one hour in 1:2 blocking buffer for near-infrared fluorescent Western blotting (Rockland, Limerick, PA, USA). The blocked membranes were incubated overnight at 4°C with primary antibodies and then washed and incubated with IR-Dye®800CW-conjugated or IR-Dye®680RD-conjugated secondary donkey anti-rabbit or anti-mouse antibodies (LI-COR Biosciences, Lincoln, NE, USA) diluted 1:15,000 in blocking buffer with 0.05% Tween 20 and 0.01% SDS.
The primary antibodies used were: anti-lamin A/C (1:2,000, 10298-1-AP, Proteintech, Manchester, UK), anti-Progerin (1/50, sc-81611, Santa Cruz Biotechnology, CA, USA), anti-NRF2 (1:2000, 80593-1-RR, Proteintech, Manchester, UK), anti-HO-1 (1:1,000, 66743-1-Ig, Proteintech, Manchester, UK), anti-KEAP1 (1:2,000, 10503-2-AP, Proteintech, Manchester, UK), anti-α-tubulin (1:4,000, T6199, Sigma Aldrich) and anti-GAPDH (1:40,000, MAB374, Millipore Millipore, Burlington, MA, USA).
All blots were imaged using a ChemiDoc MP Imaging System (Bio-Rad), and bands were quantified using Image Lab Software (Bio-Rad, Hercules, CA, USA). GAPDH or α-tubulin was used as a total cellular protein loading control.

3D structured illumination microscopy (3D-SIM)

After slide preparation (see *Material and Methods*), data were obtained on a commercial N-SIM system (Nikon) using an oil immersion objective CFI Apochromat, ×100 1.49 NA. 3D-SIM

sequences were acquired with a pixel size of 65 and 120 nm in the xy and xz plane (five phases, three angles and 15 raw images per plane). Fluorophores were excited using a 405nm laser (Hoechst), 488 nm laser (Alexa Fluor 488) and a 640 nm laser (Alexa Fluor 647) and their respective emissions collected on a sCMOS Hamamatsu ORCA-Fusion BT camera with the following emission filters EM460/25 (range 447-472 nm for Hoechst), EM525/50 (range 500-550 nm for Alexa Fluor 488) and EM700/75 (range 663-738 nm for Alexa Fluor 647). Transmitted laser power and camera exposure time were kept identical between samples and identical labels. Raw SIM data were processed and reconstructed into super-resolution images with NIS Elements.

Intensity profiling (Figure 2D): Intensity profiles of Lamin A/Progerin and NRF2 were generated using Fiji (ImageJ) *(Schindelin, J., Arganda-Carreras, I., Frise, E. et al. Fiji: an open-source platform for biological-image analysis. Nat Methods 9, 676–682 (2012). https://doi.org/10.1038/nmeth.2019)*. A 10-pixel-thick line was drawn across the nuclear periphery (dotted white rectangle, Figure 2D) to define the region of interest. Fluorescence intensity values were measured and extracted using the Plot Profile Tool. The intensity scale was kept consistent between the Control (C4) and HGPS patient (HGPS3) samples to allow direct comparison of NRF2 intensities. The peaks correspond the nuclear *lamina*, where Lamin A (in Control) and Progerin (in HGPS) show highest expression. The left side of the peak corresponds to the nucleus and the right side to the cytoplasm.

Nuclear *lamina* analysis (Figure 2D & Figure S6): The nuclear *lamina* region was selected by drawing a 20-pixel-thick line (white bracket) in Fiji (ImageJ). The curved nuclear *lamina* was straightened using the "Straighten ROI" tool for comparative analysis between control and patient cell lines. The *lamina* region expressing Lamin A or Progerin was highlighted using the mean auto-threshold function for improved visualization.

Sum intensity projection (Figure S6): To assess nuclear NRF2 levels, a sum intensity projection of 16 optical slices (1.92 µm total thickness) was generated in Fiji. The projection was centered at the nuclear equator, excluding the upper and lower slices to eliminate cytoplasmic signal contamination.

Proliferation and toxicity assays

Cell proliferation was assessed using three independent methods: (i) the CellTiter-Glo® 2.0 Cell Viability Assay, which quantifies ATP as a marker of metabolically active cells, (ii) the BrdU Cell Proliferation ELISA Kit, which measures BrdU incorporation into newly synthesized DNA, and (iii) Ki67 immunofluorescence.

Cell Viability Assays were performed as previously described (Frankel et al. 2022). Briefly, control fibroblasts were seeded at 5,000 cells per well in 96-well plates containing 100 mL DMEM. Toxicity and proliferation rates were assessed 96 h post transfection using CellTox® Green Cytotoxicity Assay and CellTiter-Glo®, respectively (Promega, Madison, WI, USA), according to the manufacturer’s instructions. The CellTox® Green Cytotoxicity Assay was included to avoid misinterpretation of CellTiter-Glo® results, since a reduction in luminescence may reflect either decreased proliferation or increased cell lysis due to toxic effects.

Fluorescence and luminescence were recorded using a GloMax® Microplate Reader (Promega).
Proliferation was further analyzed in control or patient fibroblasts 96 hours after miRNA transfection using the BrdU Cell Proliferation ELISA Kit (ab126556, Abcam, Cambridge, UK) according to the manufacturer's instructions. Negative controls included wells lacking either cells or BrdU to account for nonspecific binding. Absorbance was measured at 450 nm using the GloMax® Microplate Reader.
For Ki67 detection, immunofluorescence was performed as described in the Materials and Methods section.

Seahorse Assay

Oxygen consumption rate (OCR) was measured to assess mitochondrial function. By sequentially adding specific inhibitors, we determined following respiratory parameters:
Basal respiration was derived by subtracting non-mitochondrial respiration (OCR measured after Rot/AA addition) to basal OCR. ATP-linked respiration was calculated by subtracting OCR following oligomycin from basal OCR. The maximal respiratory capacity corresponded to OCR following FCCP injection with the deduction of non-mitochondrial respiration. At last, reserve capacity, also called mitochondrial spare respiratory capacity was calculated by subtracting basal OCR to OCR post FCCP injection (Rose et al. 2014).
ECAR is the reflect of glycolysis and was concomitantly measured with OCR. ECAR rate before oligomycin injection corresponds to basal ECAR, and glycolytic reserve capacity was evaluated by subtracting basal ECAR to ECAR following oligomycin injection (Rose et al. 2014).
In order to normalize OCR and ECAR according to cell counts, cells were immediately fixed with 4% PFA after Seahorse measurements and cell nuclei stained by Hoechst dye (final concentration 150 ng/mL, diluted in PBS (Phosphate Buffer Saline). Images of whole wells were acquired by Zeiss Axio Observer.Z1/7 inverted microscope (Oberkochen, Germany). Nuclei were segmented using StarDist algorithm, and the resulting segmentations were analyzed in QuPath to quantify cell counts for each well.
