# Supplementary material for: miR‐140‐5p Overexpression Contributes to Oxidative Stress and Mitochondrial Dysfunction in Hutchinson‐Gilford Progeria Syndrome Fibroblasts Through NRF2 Pathway
Source: Aging Cell. 2025 Oct 31;24(12):e70276. doi: 10.1111/acel.70276 (PMC12686586; doi:10.1111/acel.70276)
Supplement: Supplementary file 1 — Appendix S1: acel70276‐sup‐0001‐AppendixS1. [file ACEL-24-e70276-s001.zip › acel70276-sup-0001-AppendixS1/acel70276-sup-0009-Figure S7.pdf]

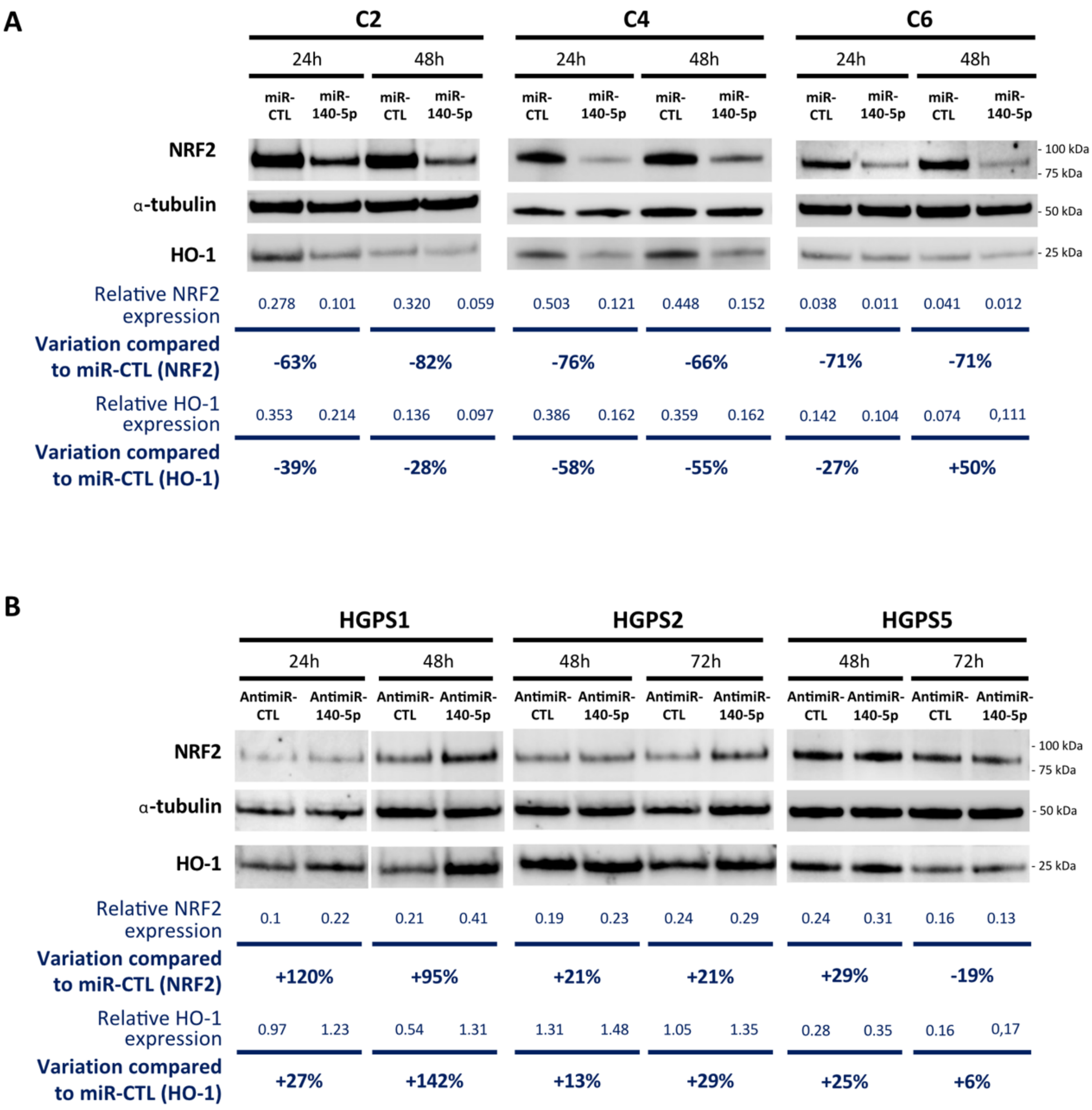

Figure S7: miR-140-5p modulation induces NRF2 variations in control (mimics) and HGPS (antimiR-140-5p) fibroblasts. (A) Western blot analysis of whole-cell lysates from 3 control cell lines (C2, C4, C6) after miR-140-5p or miR-CTL transfection at 24 and 48 hours. Detection of NRF2, HO-1, and α-tubulin protein levels. **(B)** Western blot analysis of whole-cell lysates from fibroblasts of 3 HGPS patients (HGPS1, HGPS2, HGPS5) after antimiR-140-5p or antimiR-CTL transfection at 24, 48 or 72 hours. Detection of NRF2, HO-1, and α-tubulin protein levels.
