# Supplementary material for: miR‐140‐5p Overexpression Contributes to Oxidative Stress and Mitochondrial Dysfunction in Hutchinson‐Gilford Progeria Syndrome Fibroblasts Through NRF2 Pathway
Source: Aging Cell. 2025 Oct 31;24(12):e70276. doi: 10.1111/acel.70276 (PMC12686586; doi:10.1111/acel.70276)
Supplement: Supplementary file 1 — Appendix S1: acel70276‐sup‐0001‐AppendixS1. [file ACEL-24-e70276-s001.zip › acel70276-sup-0001-AppendixS1/acel70276-sup-0013-Figure S11.pdf]

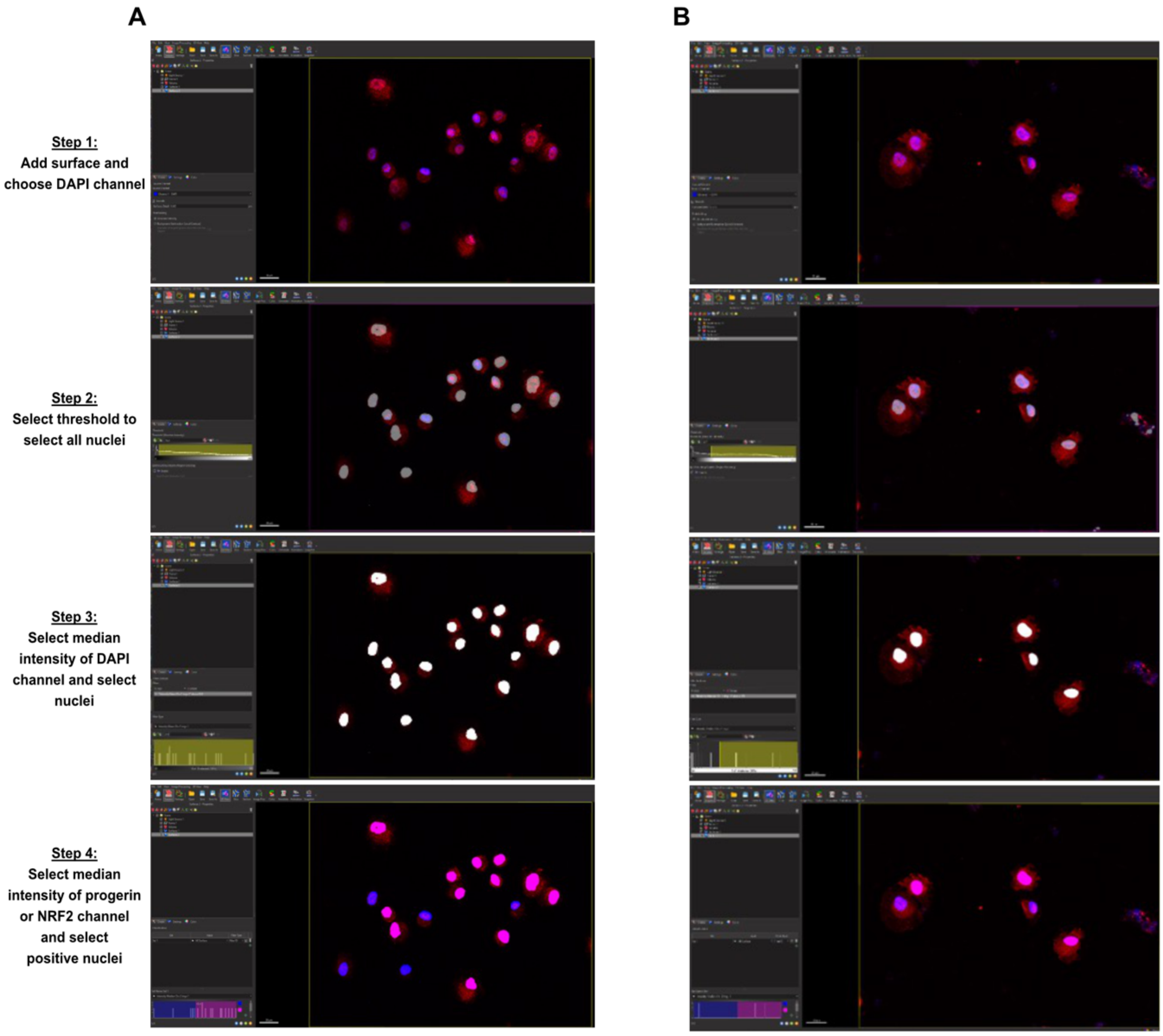

Figure S11: Illustration of the different analysis steps of sorted fibroblasts for progerin and NRF2 expression using IMARIS software. Nuclei were selected using the DAPI channel. Positive or negative nuclei for progerin or NRF2 were determined according to their median intensity.
