# Supplementary material for: miR‐140‐5p Overexpression Contributes to Oxidative Stress and Mitochondrial Dysfunction in Hutchinson‐Gilford Progeria Syndrome Fibroblasts Through NRF2 Pathway
Source: Aging Cell. 2025 Oct 31;24(12):e70276. doi: 10.1111/acel.70276 (PMC12686586; doi:10.1111/acel.70276)
Supplement: Supplementary file 1 — Appendix S1: acel70276‐sup‐0001‐AppendixS1. [file ACEL-24-e70276-s001.zip › acel70276-sup-0001-AppendixS1/acel70276-sup-0003-Figure S1.pdf]

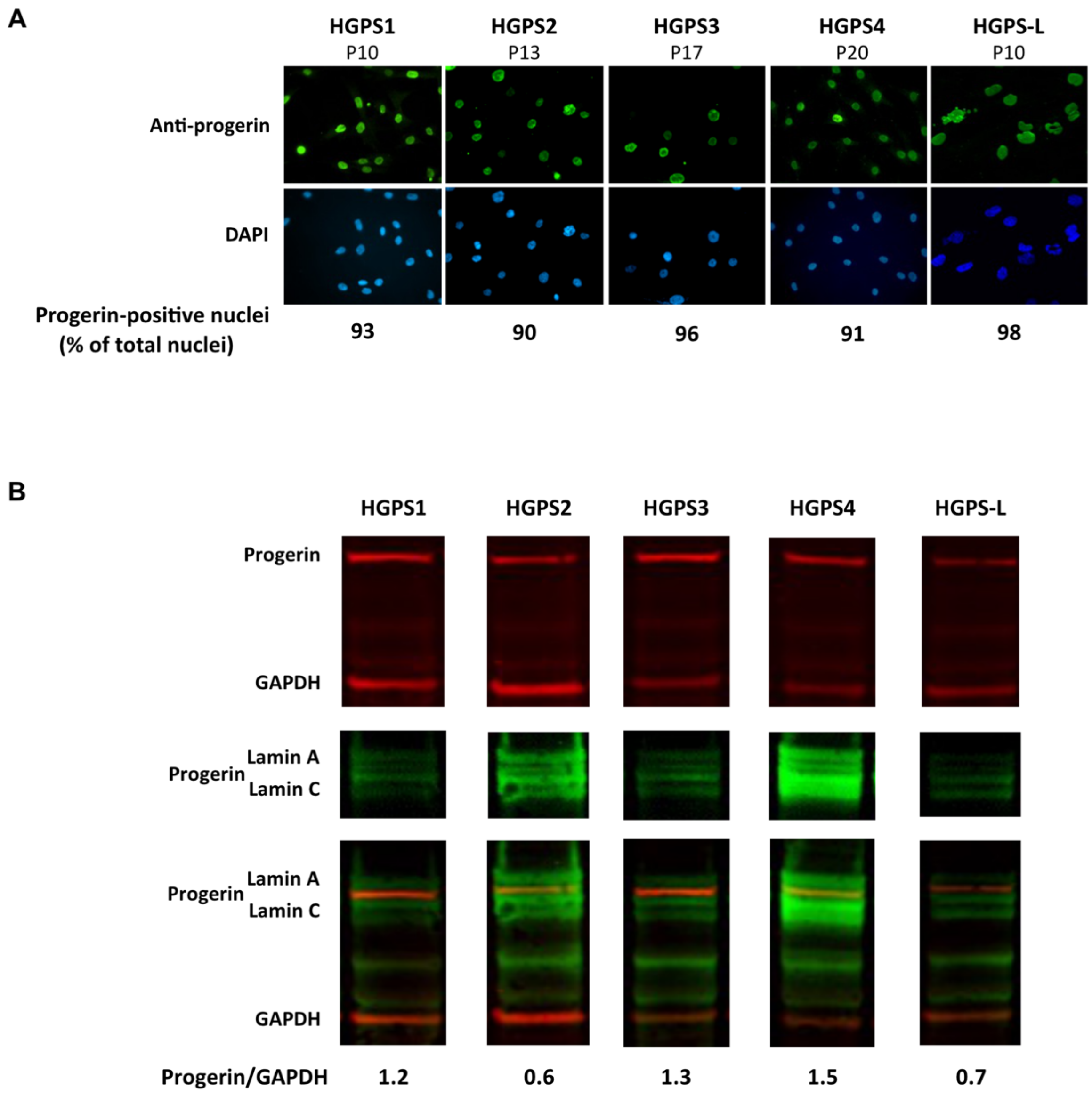

Figure S1: Selection of HGPS fibroblasts for miRNA-seq based on progerin expression. (A) Representative immunofluorescence images of HGPS (n=4, HGPS1, HGPS2, HGPS3 and HGPS4) and HGPS-like (HGPS-L) fibroblasts stained with progerin antibody (green) and counterstained with DAPI (blue). Corresponding passages are indicated for each cell line. Percentage of progerin-positive nuclei compared to total nuclei are indicated for each cell line. **(B)** Western blot analysis of whole-cell lysates from HGPS/HGPS-like fibroblasts. Detection of lamin A/C, progerin, and GAPDH. Relative progerin expression is indicated for each cell line.
