# Supplementary material for: miR‐140‐5p Overexpression Contributes to Oxidative Stress and Mitochondrial Dysfunction in Hutchinson‐Gilford Progeria Syndrome Fibroblasts Through NRF2 Pathway
Source: Aging Cell. 2025 Oct 31;24(12):e70276. doi: 10.1111/acel.70276 (PMC12686586; doi:10.1111/acel.70276)
Supplement: Supplementary file 1 — Appendix S1: acel70276‐sup‐0001‐AppendixS1. [file ACEL-24-e70276-s001.zip › acel70276-sup-0001-AppendixS1/acel70276-sup-0011-Figure S9.pdf]

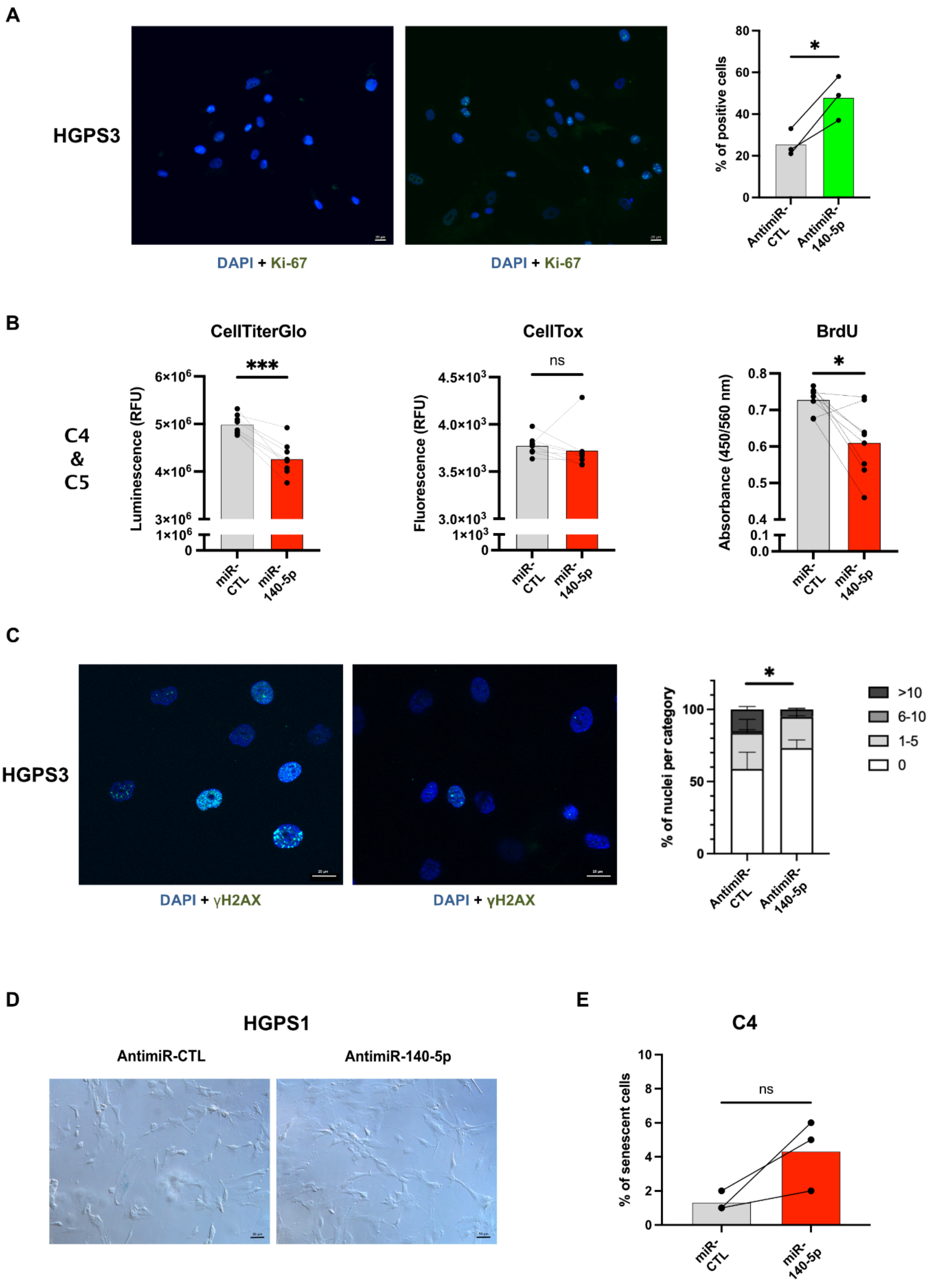

Figure S9: miR-140-5p overexpression affects several hallmarks of aging in HGPS fibroblasts. **(A)** Representative immunofluorescence images of Ki-67 (green) for HGPS fibroblasts (HGPS3) transfected with antimiR-140-5p or antimiR-control (antimiR-CTL) for 48 h. Nuclei were

counterstained with DAPI (blue). Scale bar = 20 µm. **Right:** Quantification of Ki-67-positive cells (%) 100 nuclei were counted per condition. Bars represent mean values (paired t-test, n = 3 per line; *p = 0.0344). **(B)** Cell proliferation assays in control fibroblasts (C4 and C5) transfected with miR-140-5p or miR-CTL. **Left:** CellTiter-Glo® Luminescent Cell Viability Assay. ATP luminescence measured in relative light units, RLU (paired t-test, n = 3 per line; ***p = 0.0003). **Middle:** Cytotoxicity measured with CellTox assay. Fluorescence was expressed in relative light units, RLU (Wilcoxon test, n = 3 per line; p = 0.1953, ns = not significant). **Right:** DNA replication measured by BrdU incorporation (paired t-test, n = 3 per line; p = 0.0119). **(C)** Representative immunofluorescence images of γH2AX (green) for quantification of DNA damages in HGPS fibroblasts (HGPS1) transfected with antimiR-140-5p or antimiR-CTL for 48 h. Nuclei were counterstained with DAPI (blue). Scale bar = 20 µm. Right: Quantification of the number of foci of γH2AX cell, classified into categories (0; 1–5; 6–10; >10). 100 nuclei were counted per condition (Fisher's exact test, n = 4; p = 0.0359). **(G-H)** Colorimetric detection of senescence-associated β-galactosidase. **(G)** Representative images of HGPS fibroblasts (HGPS1) transfected with antimiR-140-5p or antimiR-CTL for 48 h. Scale bar = 50 µm. **(H)** Quantification of senescent cells (%) in control fibroblasts (C4) transfected with miR-140-5p or miR-CTL for 48 h. 100 nuclei were counted per condition. Bars represent mean values (Wilcoxon test, n = 3; p = 0.250, ns = not significant).
