# Supplementary material for: miR‐140‐5p Overexpression Contributes to Oxidative Stress and Mitochondrial Dysfunction in Hutchinson‐Gilford Progeria Syndrome Fibroblasts Through NRF2 Pathway
Source: Aging Cell. 2025 Oct 31;24(12):e70276. doi: 10.1111/acel.70276 (PMC12686586; doi:10.1111/acel.70276)
Supplement: Supplementary file 1 — Appendix S1: acel70276‐sup‐0001‐AppendixS1. [file ACEL-24-e70276-s001.zip › acel70276-sup-0001-AppendixS1/acel70276-sup-0004-Figure S2.pdf]

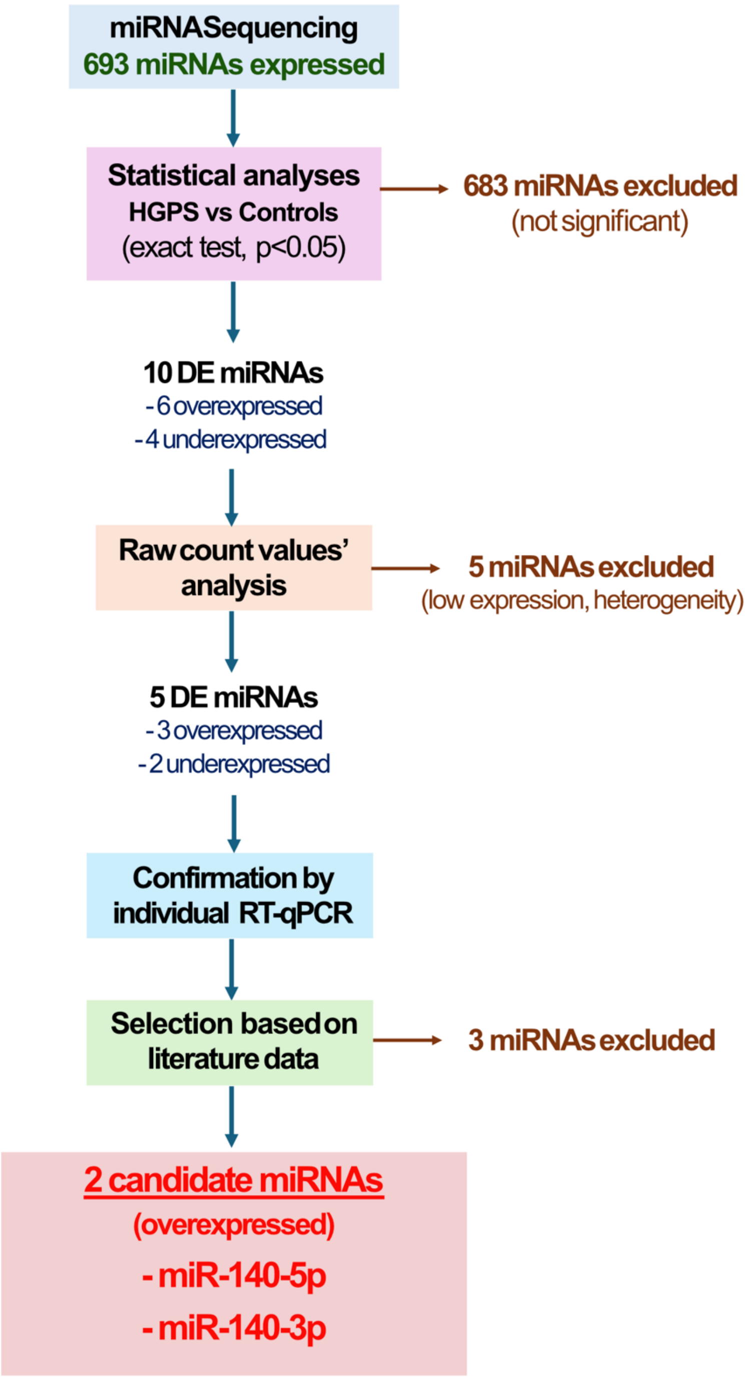

Figure S2: miRNA candidate selection pipeline. A flowchart illustrating the steps involved in selecting candidate miRNAs. Key steps include: 1) Statistical analysis to identify differentially expressed (DE) miRNAs; 2) Filtering based on expression level and homogeneity; 3) RT-qPCR validation; 4) Final selection based on literature and relevance to HGPS. miR-140-5p and miR-140-3p were finally selected after these 4 steps.
