# Supplementary material for: miR‐140‐5p Overexpression Contributes to Oxidative Stress and Mitochondrial Dysfunction in Hutchinson‐Gilford Progeria Syndrome Fibroblasts Through NRF2 Pathway
Source: Aging Cell. 2025 Oct 31;24(12):e70276. doi: 10.1111/acel.70276 (PMC12686586; doi:10.1111/acel.70276)
Supplement: Supplementary file 1 — Appendix S1: acel70276‐sup‐0001‐AppendixS1. [file ACEL-24-e70276-s001.zip › acel70276-sup-0001-AppendixS1/acel70276-sup-0012-Figure S10.pdf]

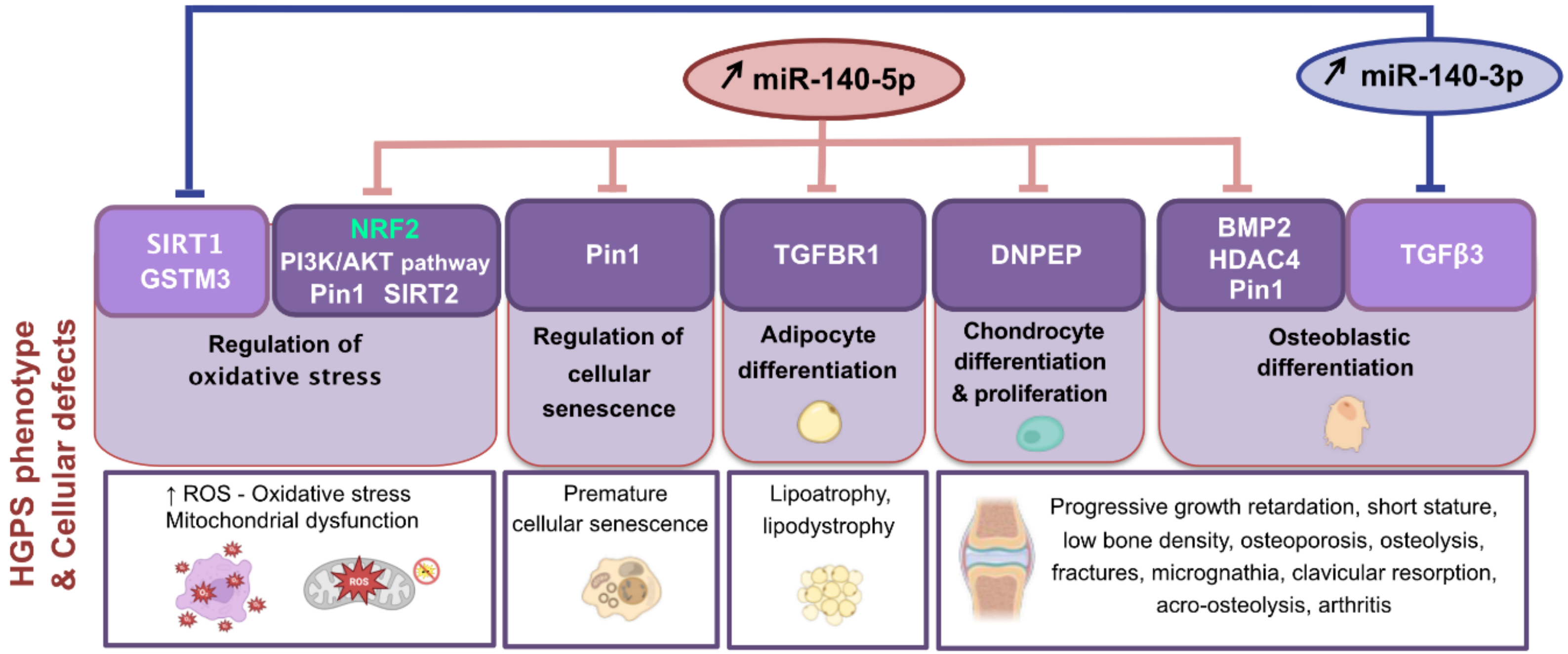


**Figure S10: A schematic representation of miR-140-5p and miR-140-3p target genes and their potential involvement in cellular processes relevant to the HGPS phenotype.** Proteins corresponding to mRNA targets (top), potentially affected cellular mechanisms and relation with phenotype (bottom) are indicated.
