# Supplementary material for: miR‐140‐5p Overexpression Contributes to Oxidative Stress and Mitochondrial Dysfunction in Hutchinson‐Gilford Progeria Syndrome Fibroblasts Through NRF2 Pathway
Source: Aging Cell. 2025 Oct 31;24(12):e70276. doi: 10.1111/acel.70276 (PMC12686586; doi:10.1111/acel.70276)
Supplement: Supplementary file 1 — Appendix S1: acel70276‐sup‐0001‐AppendixS1. [file ACEL-24-e70276-s001.zip › acel70276-sup-0001-AppendixS1/acel70276-sup-0007-Figure S5.pdf]

A

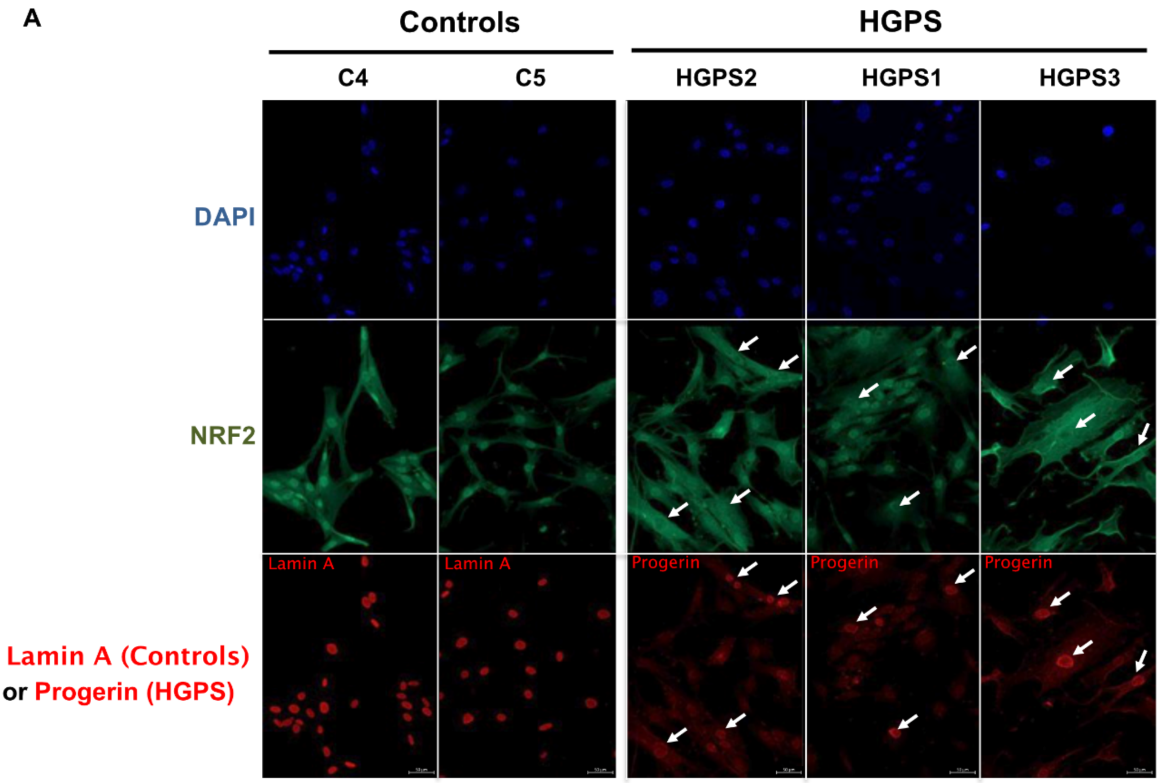

B

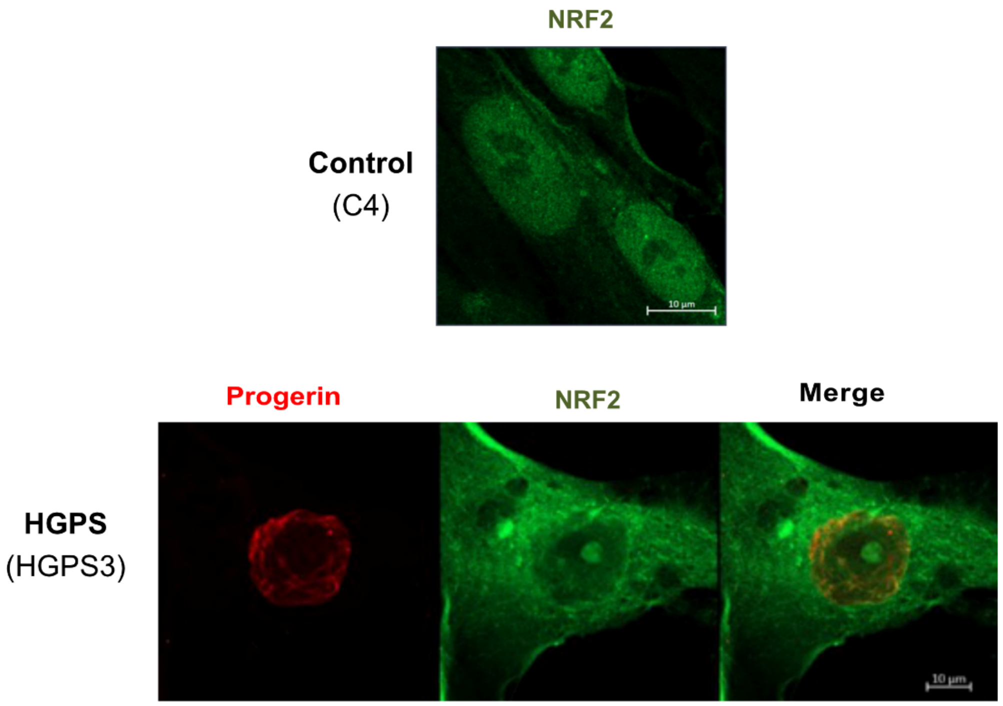

Figure S5: Nuclear NRF2 loss in HGPS fibroblasts with high progerin accumulation. (A) Representative immunofluorescence images of control (C4 and C5) and HGPS fibroblasts (HGPS1, HGPS2 and HGPS3) stained for NRF2 and lamin A/C (control) or progerin (HGPS) and

DAPI. Arrows indicate nuclei expressing progerin but not NRF2. Scale bar = 50 µm. **(B)** Representative confocal immunofluorescence images of control (C4) and HGPS fibroblasts (HGPS3) stained for NRF2 and progerin for HGPS. Scale bar=10 µm.
