# Supplementary material for: miR‐140‐5p Overexpression Contributes to Oxidative Stress and Mitochondrial Dysfunction in Hutchinson‐Gilford Progeria Syndrome Fibroblasts Through NRF2 Pathway
Source: Aging Cell. 2025 Oct 31;24(12):e70276. doi: 10.1111/acel.70276 (PMC12686586; doi:10.1111/acel.70276)
Supplement: Supplementary file 1 — Appendix S1: acel70276‐sup‐0001‐AppendixS1. [file ACEL-24-e70276-s001.zip › acel70276-sup-0001-AppendixS1/acel70276-sup-0005-Figure S3.pdf]

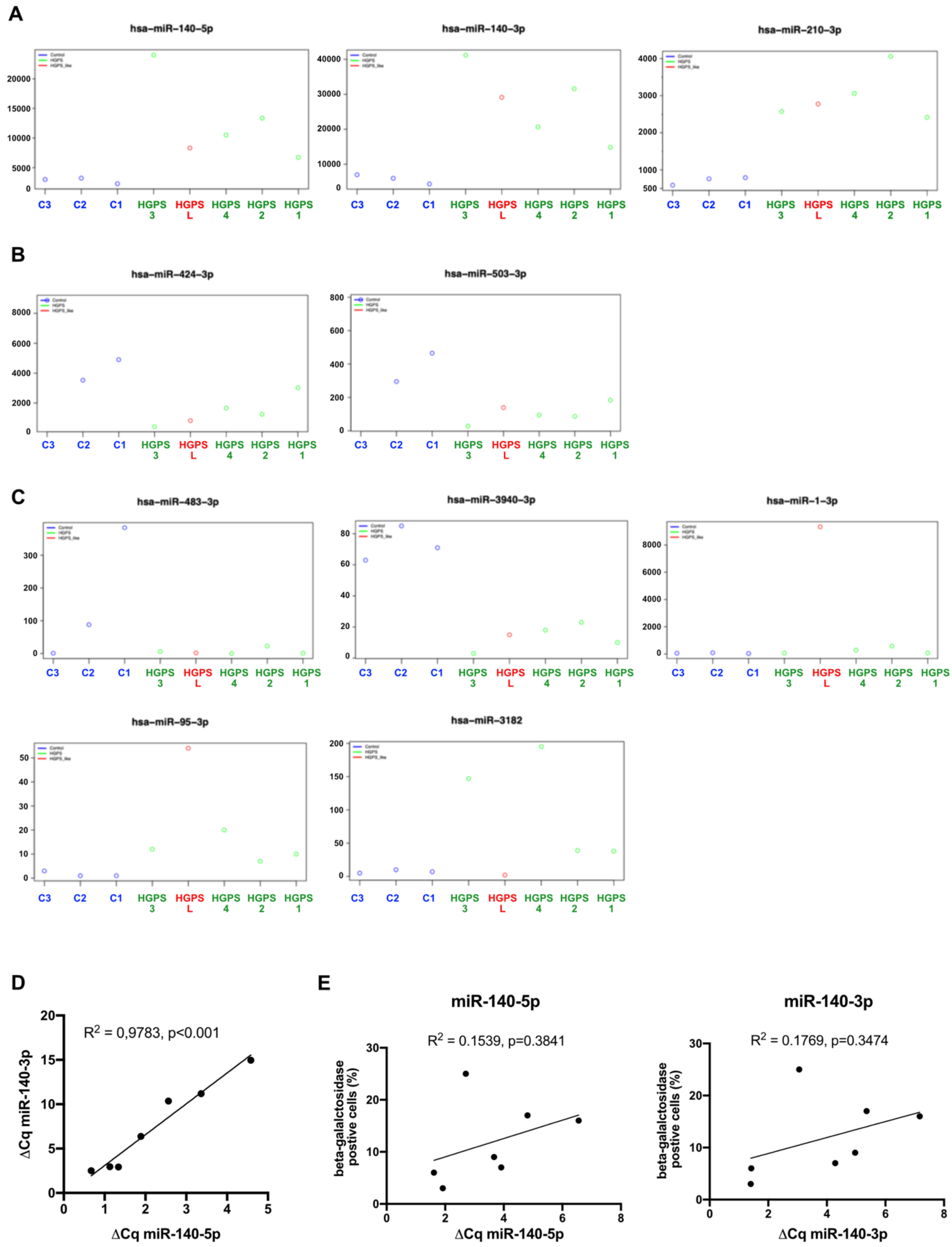

Figure S3: Selection of miR-140-5p and miR-140-3p and correlation of their expression with senescence. (A-C) Raw count plots of differentially expressed miRNAs. miRNAs were selected based on expression level (>200 counts) and homogeneity within groups (control and HGPS/HGPS-like). **(A)** Selected upregulated miRNAs in HGPS fibroblasts (n=3). **(B)** Selected downregulated miRNAs in HGPS fibroblasts (n=2). **(C)** miRNAs excluded for the next step. **(D)**

Correlation graph between miR-140-5p and miR-140-3p expression (ΔCq values) (n=7). Pearson correlation coefficient (R^2) and p-value are shown. **(E)** Correlation graphs between miR-140-5p or miR-140-3p expression (ΔCq values) and senescence (percentage of β-galactosidase-positive cells). Pearson correlation coefficient (R^2) and p-value are shown.
