# Supplementary material for: miR‐140‐5p Overexpression Contributes to Oxidative Stress and Mitochondrial Dysfunction in Hutchinson‐Gilford Progeria Syndrome Fibroblasts Through NRF2 Pathway
Source: Aging Cell. 2025 Oct 31;24(12):e70276. doi: 10.1111/acel.70276 (PMC12686586; doi:10.1111/acel.70276)
Supplement: Supplementary file 1 — Appendix S1: acel70276‐sup‐0001‐AppendixS1. [file ACEL-24-e70276-s001.zip › acel70276-sup-0001-AppendixS1/acel70276-sup-0010-Figure S8.pdf]

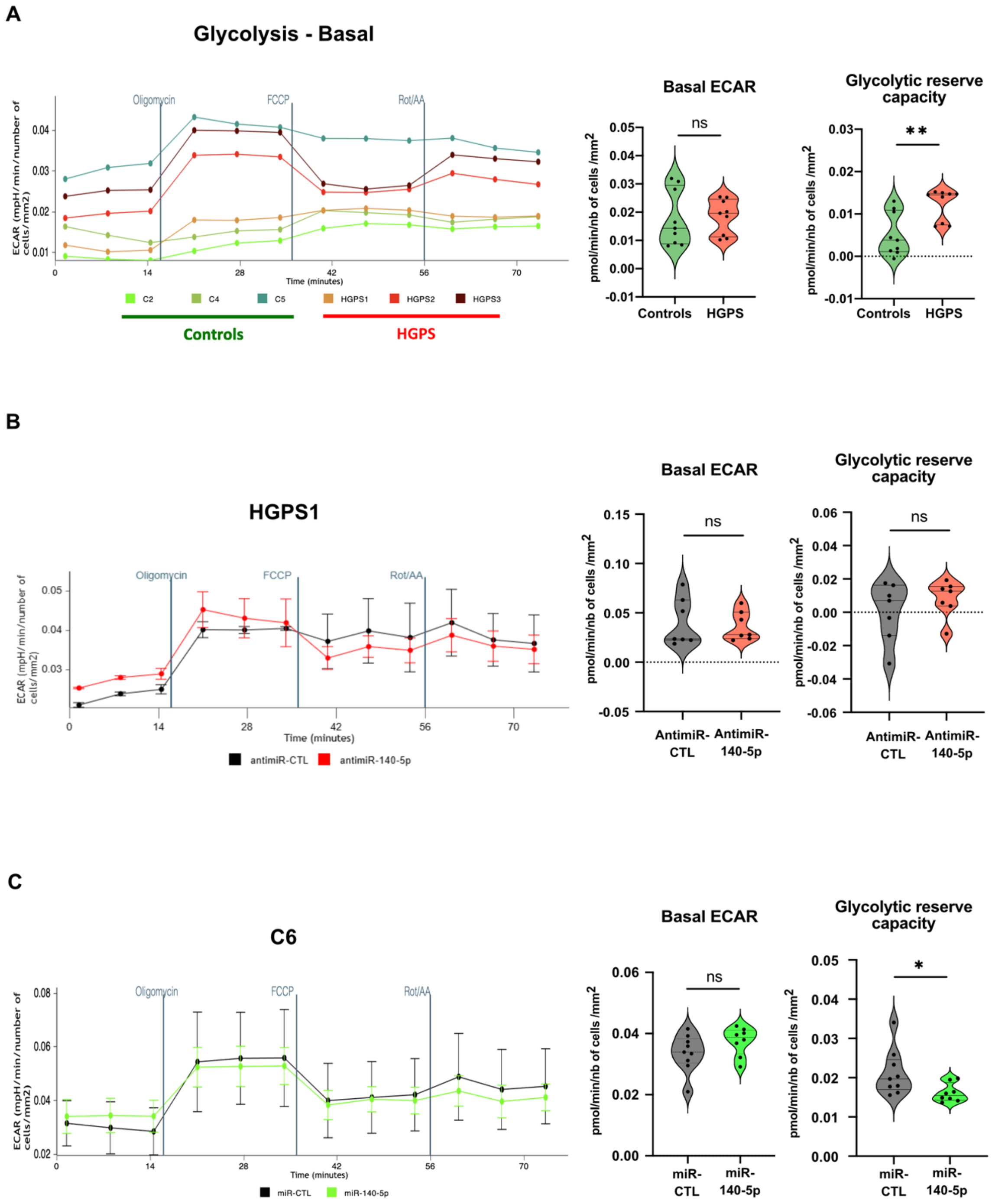

Figure S8: miR-140-5p overexpression leads to glycolysis modification in HGPS fibroblasts. (A) Seahorse assay comparing ECAR levels between control (C2, C4 and C5) and HGPS (HGPS1, HGPS2 and HGPS3) fibroblasts. The graphs on the right compare basal ECAR and glycolytic reserve capacity. Violin plots show the distribution of ECAR across samples (unpaired t-test or Mann-Whitney test; ns: not significant, $*p \leq 0.05$, $**p < 0.01$, 3 repeated ECAR were measured per condition)**. (B)** Representative Seahorse assay showing the effect antimiR-140-5p transfection on ECAR measurement in HGPS cells (HGPS1). The graphs on the right compare basal ECAR and glycolytic reserve capacity. Violin plots show the distribution of ECAR across

samples (Mann-Whitney test; ns: not significant, n=3 cell lines (HGPS1, HGPS3 and HGPS5), n=2 or 3 experiments per condition)**. (C)** Representative Seahorse assay showing the effect of antimiR-140-5p transfection on ECAR measurement in control cells (C6). The graphs on the right compare basal ECAR and glycolytic reserve capacity. Violin plots show the distribution of ECAR across samples (unpaired t-test or Mann-Whitney test, ns: not significant, *$p \leq 0.05$, n= 3 experiments per condition).
