# Supplementary material for: miR‐140‐5p Overexpression Contributes to Oxidative Stress and Mitochondrial Dysfunction in Hutchinson‐Gilford Progeria Syndrome Fibroblasts Through NRF2 Pathway
Source: Aging Cell. 2025 Oct 31;24(12):e70276. doi: 10.1111/acel.70276 (PMC12686586; doi:10.1111/acel.70276)
Supplement: Supplementary file 1 — Appendix S1: acel70276‐sup‐0001‐AppendixS1. [file ACEL-24-e70276-s001.zip › acel70276-sup-0001-AppendixS1/acel70276-sup-0006-Figure S4.pdf]

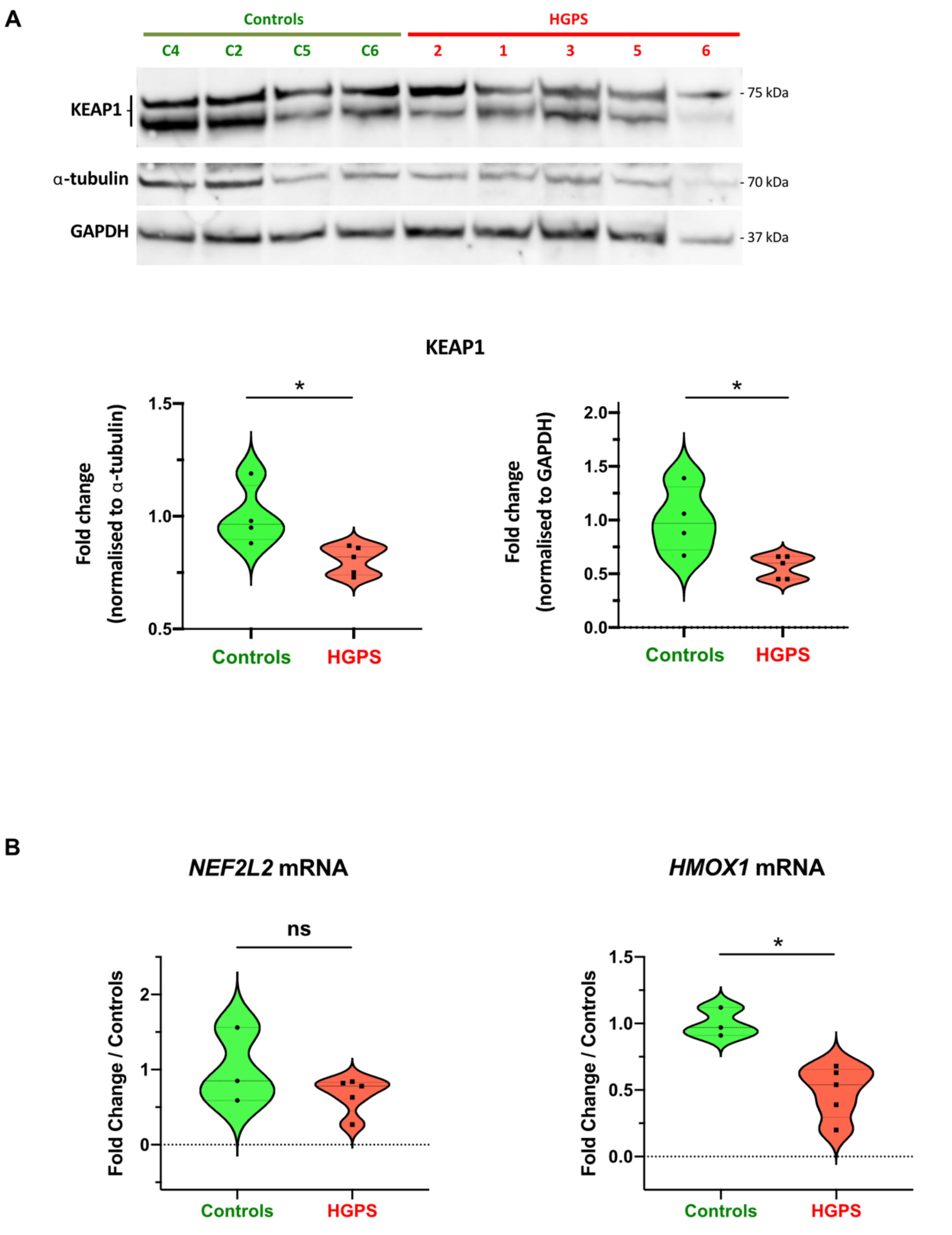

Figure S4: Potential impact of miR-140-5p overexpression on KEAP1, NRF2, and HO-1. (A) Western blot analysis of whole-cell lysates from controls and HGPS fibroblasts. Detection of KEAP1, α-tubulin, and GAPDH. Statistical analysis shows the fold change of KEAP1 expression in HGPS (n=5) compared to controls (n=4) after normalization (Mann-Whitney test, $*p \leq 0.05$).

(B) *NEF2L2* mRNA and *HMOX1* mRNA expression quantified by RT-qPCR in HGPS (n=5) compared to controls (n=3) (Mann-Whitney test, two-tailed; *NEF2L2* mRNA: p=0.3929, ns: not significant; *HMOX1* mRNA: ∗p=0.0357).
