# Supplementary material for: miR‐140‐5p Overexpression Contributes to Oxidative Stress and Mitochondrial Dysfunction in Hutchinson‐Gilford Progeria Syndrome Fibroblasts Through NRF2 Pathway
Source: Aging Cell. 2025 Oct 31;24(12):e70276. doi: 10.1111/acel.70276 (PMC12686586; doi:10.1111/acel.70276)
Supplement: Supplementary file 1 — Appendix S1: acel70276‐sup‐0001‐AppendixS1. [file ACEL-24-e70276-s001.zip › acel70276-sup-0001-AppendixS1/acel70276-sup-0008-Figure S6.pdf]

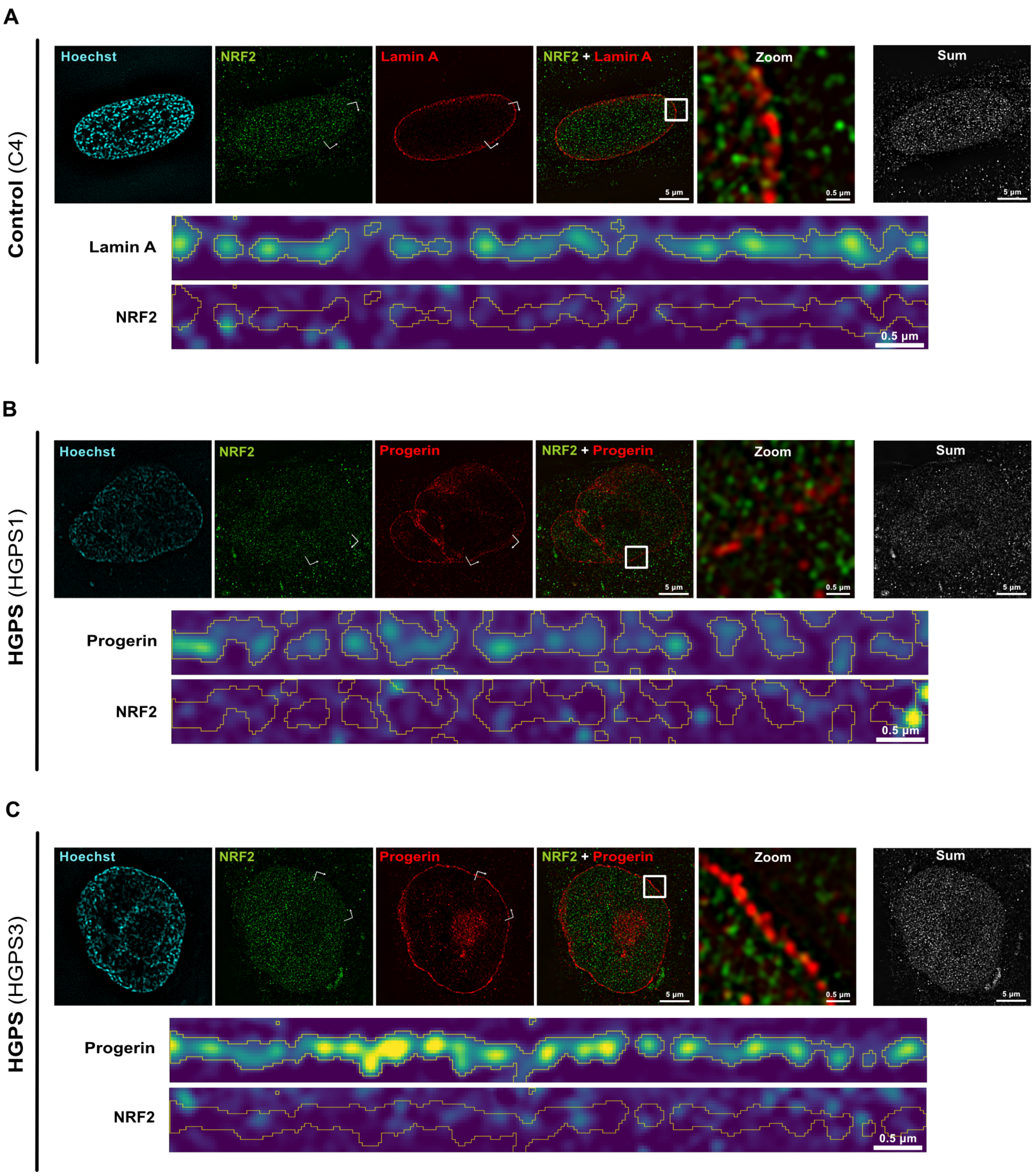

Figure S6: High-resolution microscopy analysis of NRF2 expression and localization. Representative 3D-SIM images of **(A)** control (C4), **(B)** HGPS1, and **(C)** HGPS3 patient nuclei. Nuclei are labeled with NRF2 (green), Lamin A/Progerin (red), and counterstained with Hoechst (blue). Each image shows a single optical slice at the nuclear equator, extracted from a full z-stack acquisition. The sum intensity projection represents the sum of 16 optical slices (1.92 µm total thickness). The white square highlights the magnified region (Zoom) and the white bracket marks the region of the straightened nuclear lamina (shown below the corresponding images). All scale bars are indicated in the corresponding images. Full image acquisition and analysis procedures are described in the *Supplemental Methods*.
